# Supplementary material for: Structure and reactivity of germylene-bridged digold complexes
Source: Nat Commun. 2022 Apr 4;13:1785. doi: 10.1038/s41467-022-29476-1 (PMC8979951; doi:10.1038/s41467-022-29476-1)
Supplement: Supplementary file 1 — Supplementary Information [file 41467_2022_29476_MOESM1_ESM.pdf]

Supplementary Information For

Structure and reactivity of germylene-bridged digold complexes

Wang et al.

Table of Contents

Experimental Section S2
a) VT NMR spectra and chemical shift of **3b** S2
b) Crystallographic details S5
c) UV-visible spectroscopy S10
d) ^{1}H, ^{13}C, ^{29}Si and ^{31}P Spectra of products S12
Theoretical calculations S35

Experimental Section

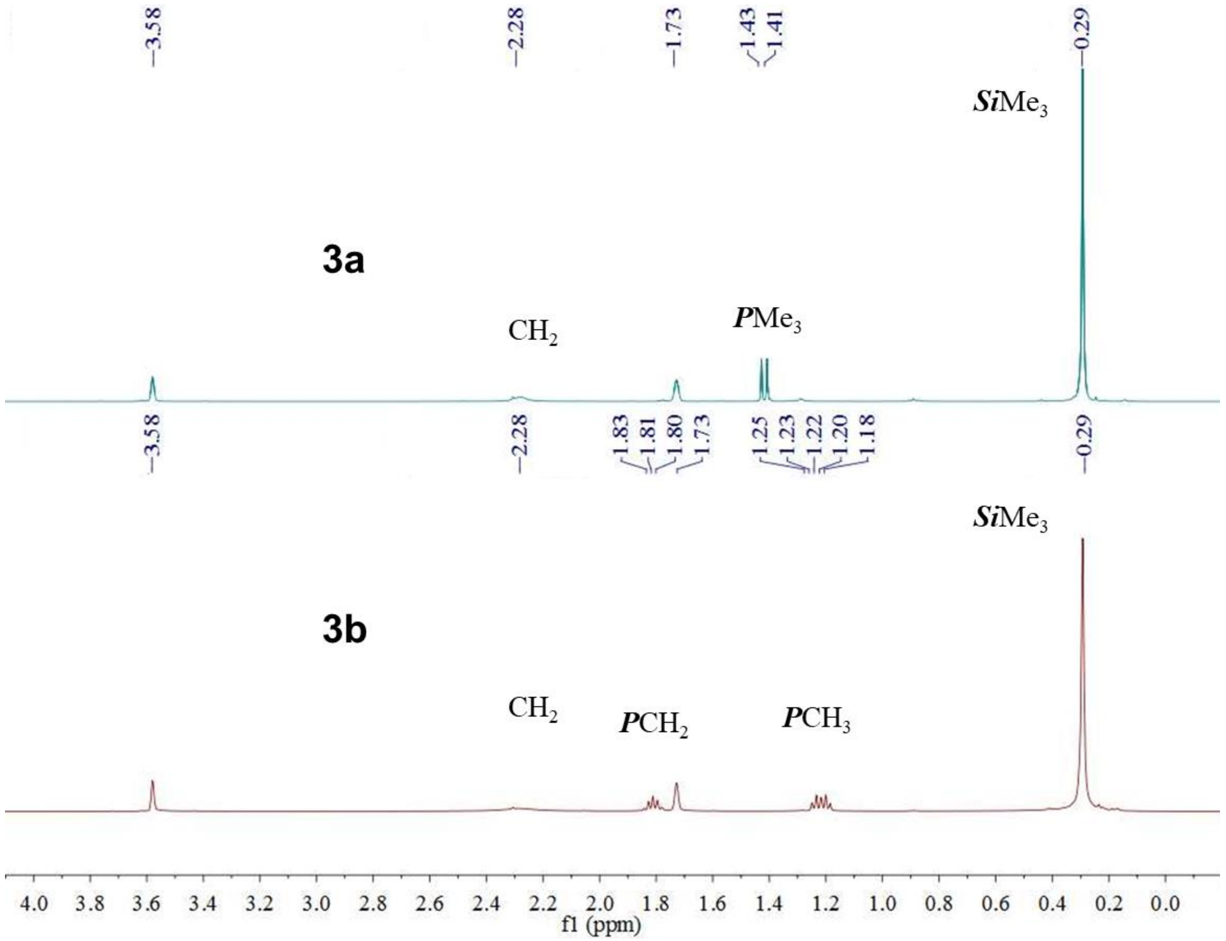

Supplementary Figure 1. ^{1}H NMR spectra of **3a** (top) and **3b** (bottom) in THF-d_8 at room temperature.

a) *VT NMR spectra and chemical shift of **3b***

The ^{1}H NMR spectra of **3b** were recorded in THF-d_8 (0.018 M). Peak positions were picked by fitting in MestReNova software.

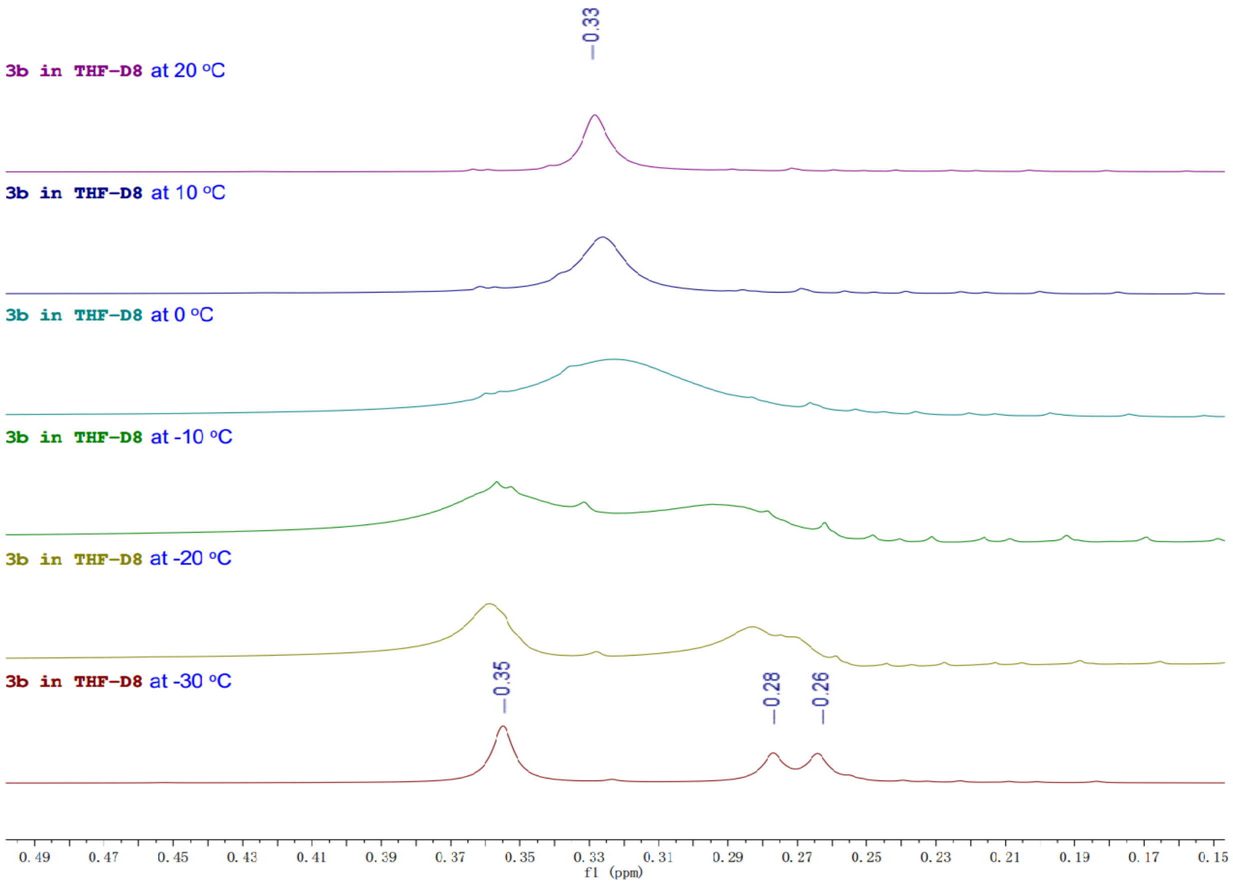

Supplementary Figure 2. ^{1}H NMR spectra of **3b** at variable temperatures from 20 ºC to –30 ºC.

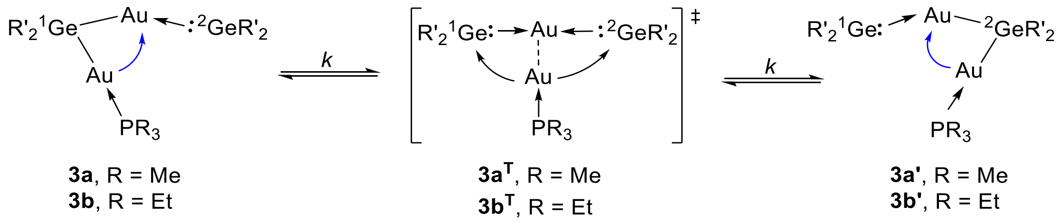

Supplementary Figure 3. Facile isomerization between **3** and its equivalent structure **3'**.

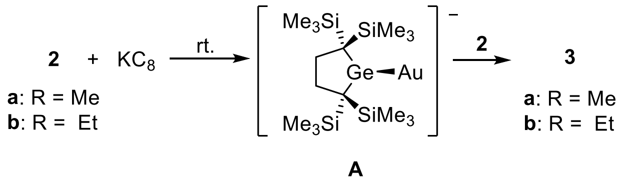

Supplementary Figure 4. A proposed mechanism for the formation of **3**

Mechanistic proposal for the cyclotrimerization of aryl isocyanates

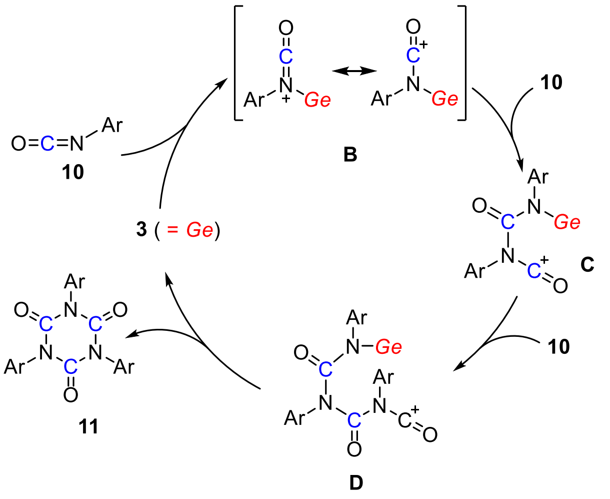

Supplementary Figure 5. A catalytic cycle proposed for a cyclic trimerization of aryl isocyanates catalyzed by **3a**.

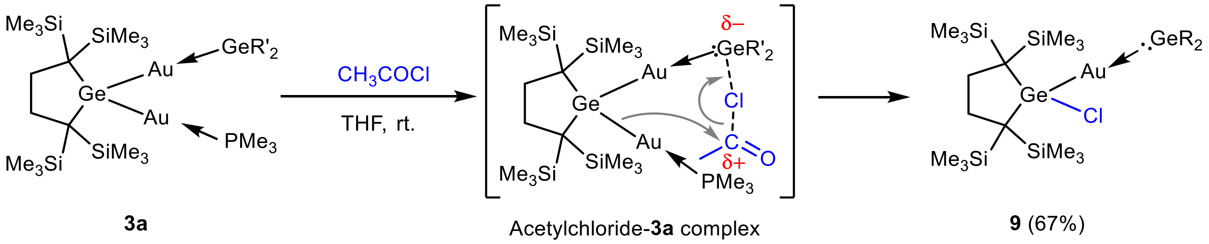

Supplementary Figure 6. Proposed reaction pathway for the formation of **9**.

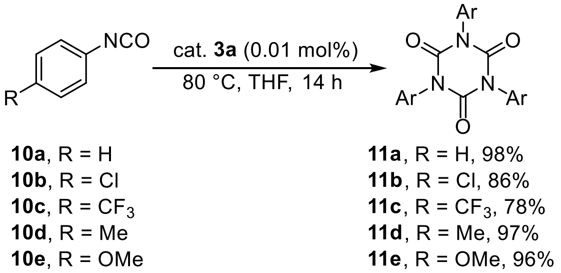

Supplementary Figure 7. Cyclic trimerization of aryl isocyanates **10** catalyzed by digoldgermane **3a**.

b) *Crystallographic details*

X-ray data collection and structural refinement. Intensity data for compounds **2b**, **3a, 3b**, **4, 5, 7** and **9** were collected using a Bruker APEX II diffractometer. The structure was solved by direct phase determination (SHELX-2014) and refined for all data by full-matrix least-squares methods on F^2. [S1,S2] All non-hydrogen atoms were subjected to anisotropic refinement. The hydrogen atoms were located at calculated positions or found in the *F*map. CCDC: 2040233, 2040232, 2040235, 2040234, 2093574, 2040236 and 2093575 contains the supplementary crystallographic data for this paper. The data can be obtained free of charge from the Cambridge Crystallography Data Center via www.ccdc.cam.ac.uk/data_reCquest/cif.

Supplementary Table 1. X-ray data for **2b**, **3a**, **3b, 4, 5, 7** and **9**.

Compounds	**2b**	**3a**	**3b**
Formula	$C_{22}H_{55}AuClGePSi_4$	$C_{35}H_{89}Au_2Ge_2PSi_8$	$C_{38}H_{95}Au_2Ge_2PSi_8$
Fw	767.99	1304.86	1346.94
Crystsyst	Triclinic	Triclinic	Monoclinic
Space group	P-1	P-1	$P2_1/n$
Size (mm^3) space group	0.21 x 0.15 x 0.15	0.36×0.35×0.29	0.25 × 0.25 × 0.20
T/K	296(2)	100.04	140(2)
a, Å	9.9896(5)	11.3169(6)	16.7554(8)
b, Å	11.5379(6)	13.4458(10)	18.3718(9)
c, Å	16.6794(9)	19.2778(14)	18.6768(8)
α, deg	71.5790(10)	83.444(2)	90
β, deg	84.8400(10)	79.181(2)	98.4697(12)
γ, deg	67.2130(10)	70.518(2)	90
V, A^3	1680.43(15)	2712.1(3)	5686.5(5)
Z	2	2	4
d_{calcd} g·cm^{-3}	1.518	1.598	1.573
μ, mm^{-1}	5.534	6.719	6.411
Refl collected	34666	48541	65807
N_{measd}	8364	16706	13544
[R int]	0.0346	0.0612	0.0602
R [I>2sigma(I)]	0.0336	0.0386	0.0489
R_w [I>2sigma(I)]	0.0925	0.0951	0.1106
GOF	1.032	1.013	1.034
Largest diff. peak/ hole [e. $Å^{-3}$]	0.957 and -1.529	2.71 and -1.87	9.306 and -1.674

Compounds	**4**	**5**
Formula	$Au_2C_{22}GeH_{58}P_2Si4$	$C_{33}H_{83}AuGe_2Si_8$
Fw	946.16	1046.86
Crystsyst	tetragonal	monoclinic
Space group	$I4_1/acd$	Cc
Size (mm^3) space group	0.31 × 0.25 × 0.18	0.34 × 0.31 × 0.24
T/K	100	100
a, Å	22.2649(7)	23.1510(12)
b, Å	22.2649(7)	16.8799(9)
c, Å	34.6740(16)	12.6367(7)
α, deg	90	90
β, deg	90	96.917(2)
γ, deg	90	90
V, A^3	17188.8(13)	4902.3(5)
Z	16	4
d_{calcd} $g \cdot cm^{-3}$	1.756	1.418
μ, mm^{-1}	15.335	4.420
Refl collected	52505	66852
N_{measd}	9056	15149
[R int]	0.0907	0.0587
R [I>2sigma(I)]	0.0658	0.0434
R_w [I>2sigma(I)]	0.0669	0.0844
GOF	1.142	1.053
Largest diff. peak/ hole [e. $Å^{-3}$]	4.63 and -1.96	1.32 and -0.81

Compounds	**7**	**9**
Formula	$C_{59}H_{109}Au_2ClGe_2P_2Si_8$	$C_{32}H_{80}AuClGe_2Si_8$
Fw	1679.68	1067.27
Crystsyst	Monoclinic	monoclinic
Space group	$P2_1/n$	$P2_1/n$
Size (mm^3) space group	0.15 × 0.15 × 0.12	0.14 × 0.12 × 0.08
T/K	150	150
a, Å	11.4018(10)	15.6807(10)
b, Å	28.876(3)	19.0646(15)
c, Å	22.400(2)	17.9997(11)
α, deg	90	90
β, deg	92.372(2)	114.697(2)
γ, deg	90	90
V, A^3	7368.7(12)	4888.7(6)
Z	4	4
d_{calcd} g·cm^{-3}	1.514	1.450
μ, mm^{-1}	5.020	9.508
Refl collected	62304	44666
N_{measd}	19836	8968
[R int]	0.0671	0.0830
R [I>2sigma(I)]	0.0392	0.0621
R_w [I>2sigma(I)]	0.0847	0.1547
GOF	1.00	1.078
Largest diff. peak/ hole [e. Å^{-3}]	1.68 and -1.38	8.41 and -2.63

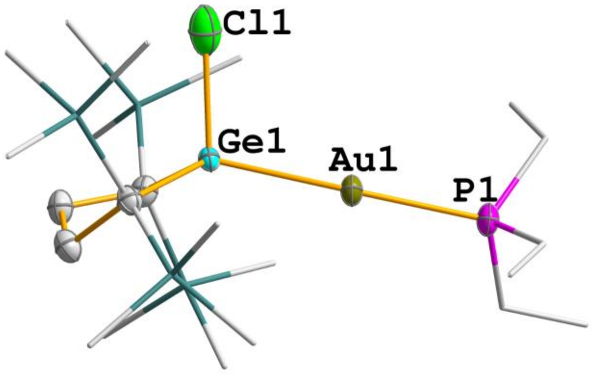

Supplementary Figure 8. Solid structure of **2b**. Thermal ellipsoids are shown at the 30% probability level. Hydrogen atoms are omitted for clarity. Trimethylsilyl, ethyl, and methyl groups are depicted in a wireframe model.

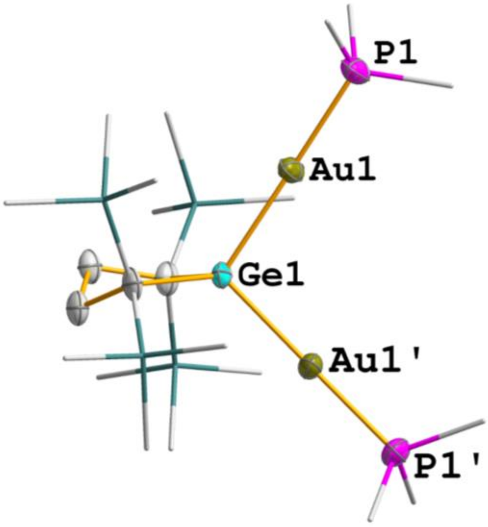

Supplementary Figure 9. Solid structure of **4**. Thermal ellipsoids are shown at the 30% probability level. Hydrogen atoms are omitted for clarity. Trimethylsilyl, ethyl, and methyl groups are depicted in a wireframe model.

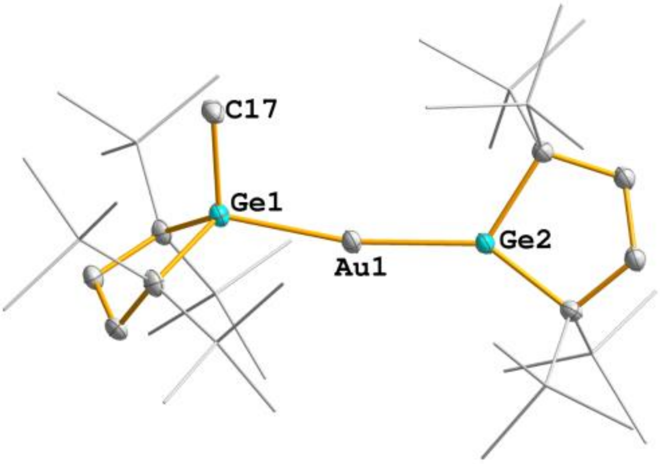

Supplementary Figure 10. Molecular structure of **5**. Hydrogen atoms are omitted for clarity. Thermal ellipsoids are shown at the 50% probability level. Trimethylsilyl, ethyl, and methyl groups are depicted in a wireframe model.

c) *UV-visible spectroscopy*

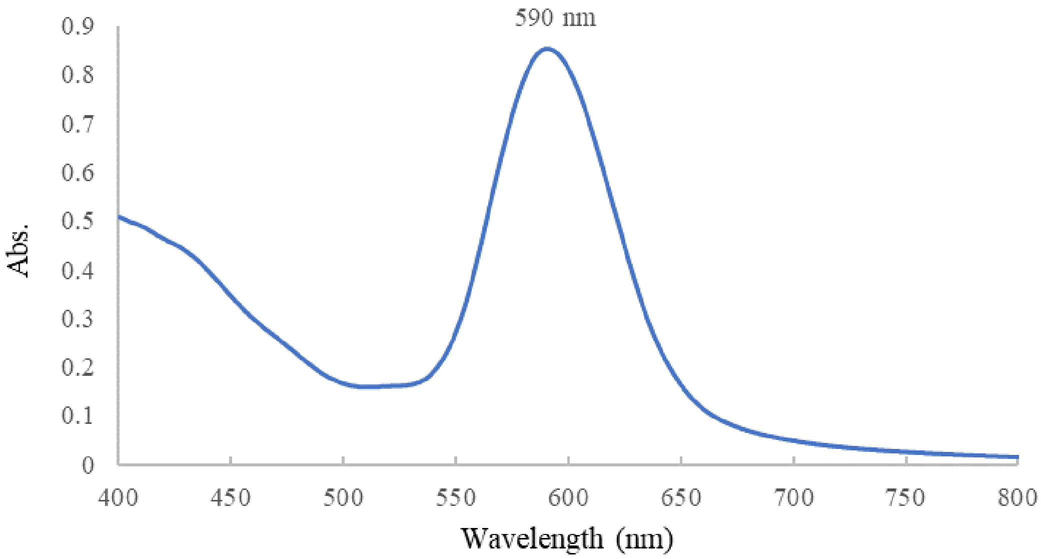

Supplementary Figure 11. UV-visible spectrum of compound **3a** in THF at 25 ºC (ε = 3558 M^{-1}•cm^{-1})

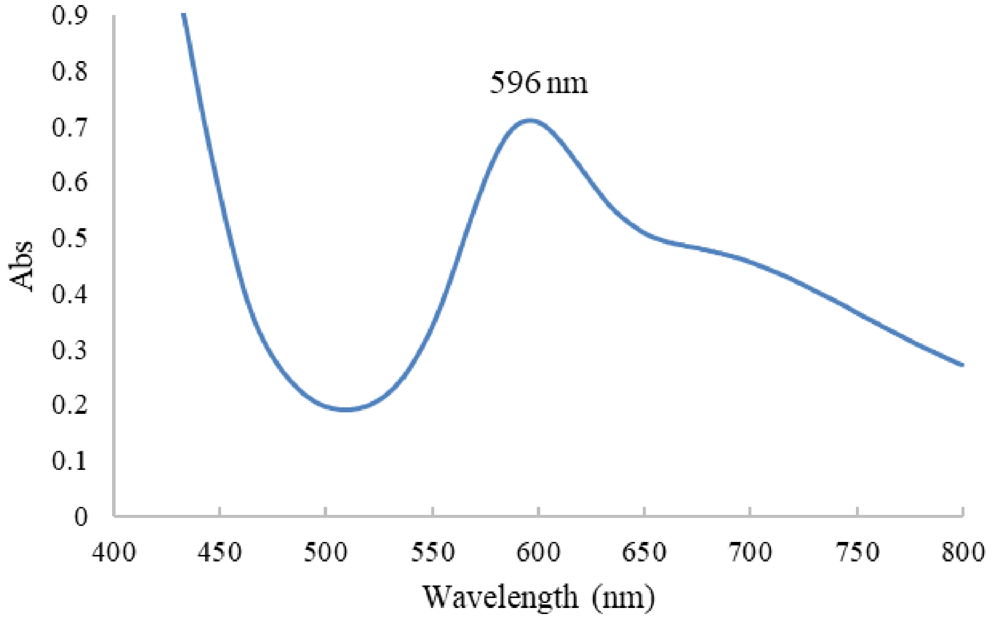

Supplementary Figure 12. UV-visible spectrum of compound **3b** in THF at 25 ºC (ε = 3556 M^{-1}•cm^{-1})

d) ^{1}H, ^{13}C, ^{29}Si and ^{31}P Spectra of products

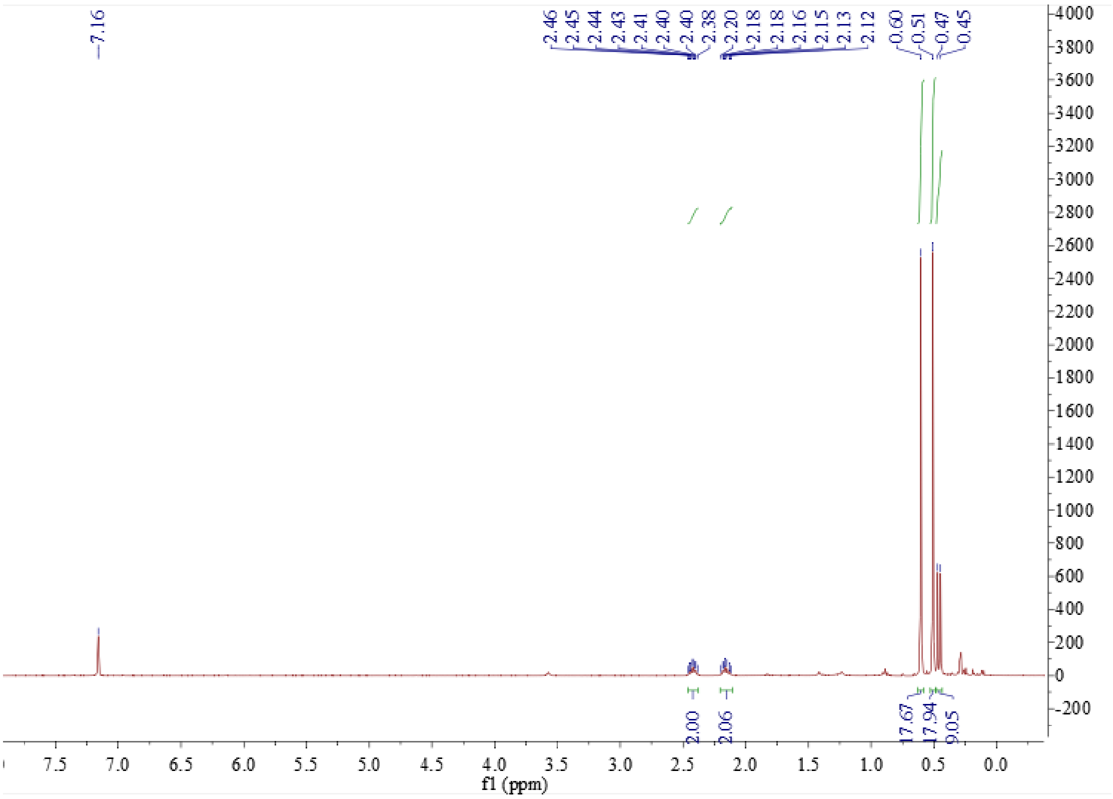

Supplementary Figure 13. ^{1}H NMR spectrum of **2a** in benzene-d_6 at 25 °C

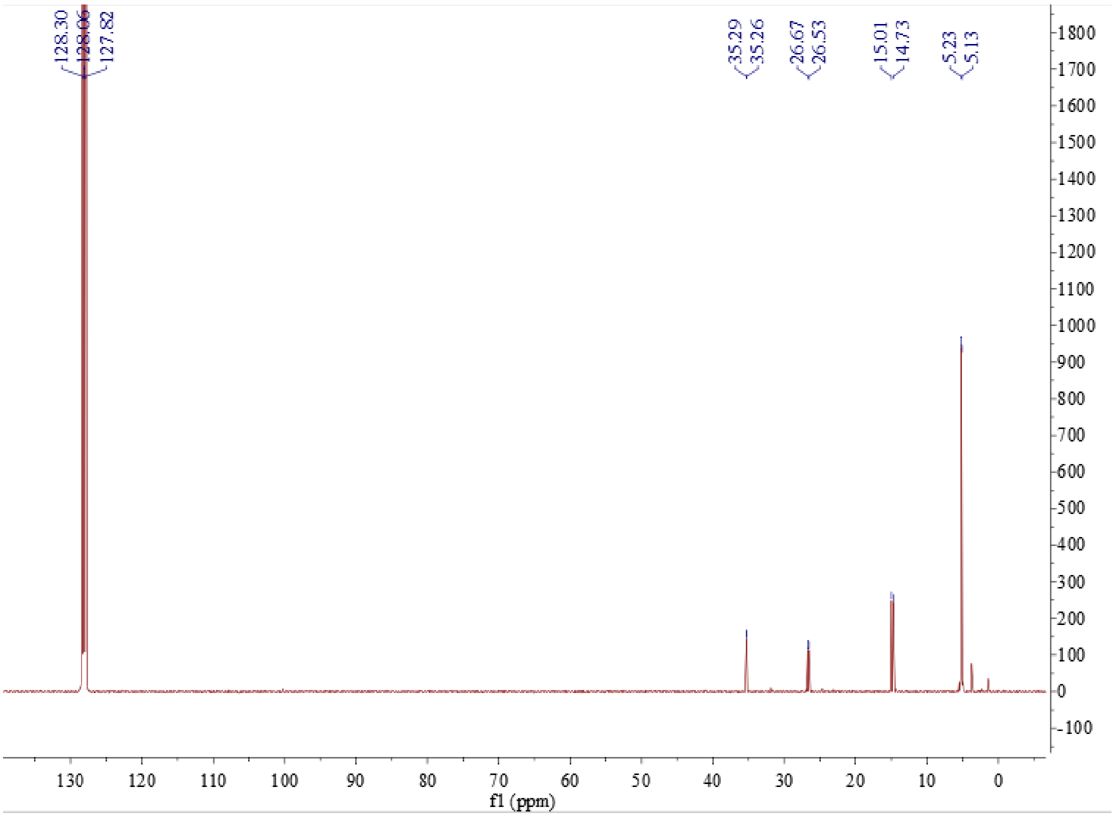

Supplementary Figure 14. ^{13}C NMR spectrum of **2a** in benzene-d_6 at 25 °C

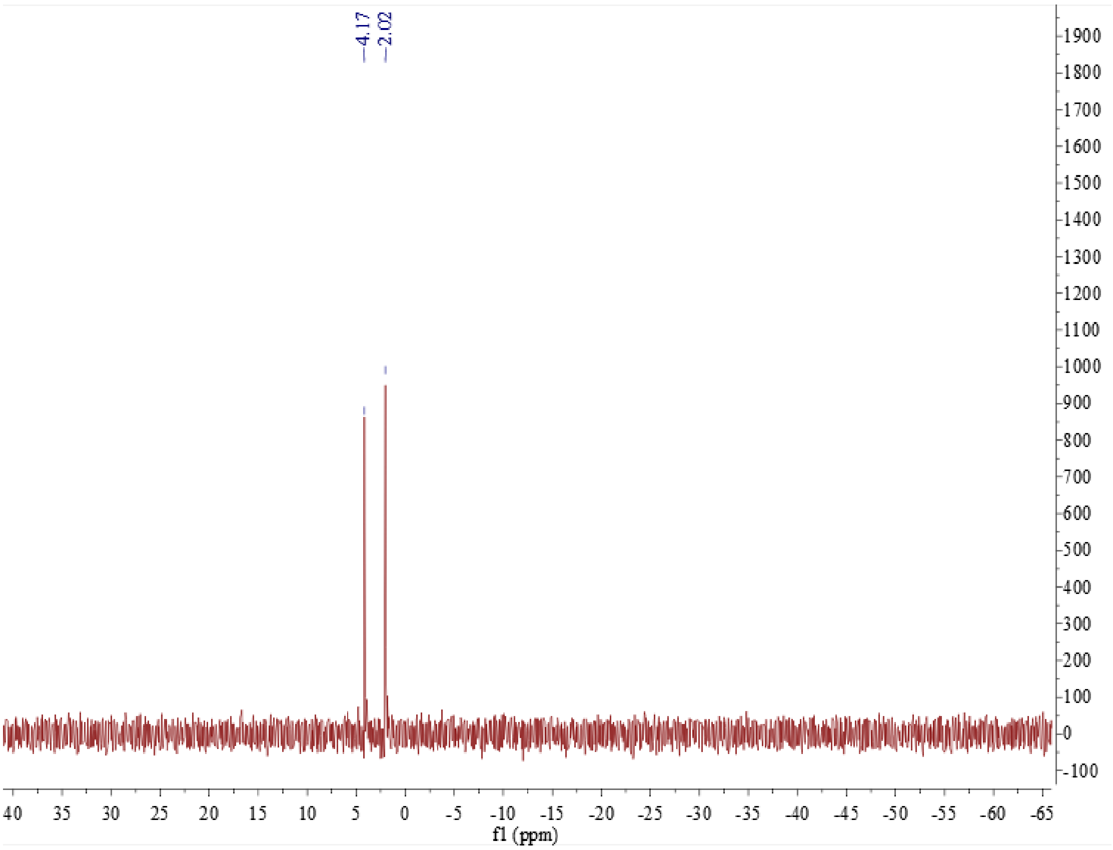

Supplementary Figure 15. ^{29}Si NMR spectrum of **2a** in benzene-d_6 at 25 ºC

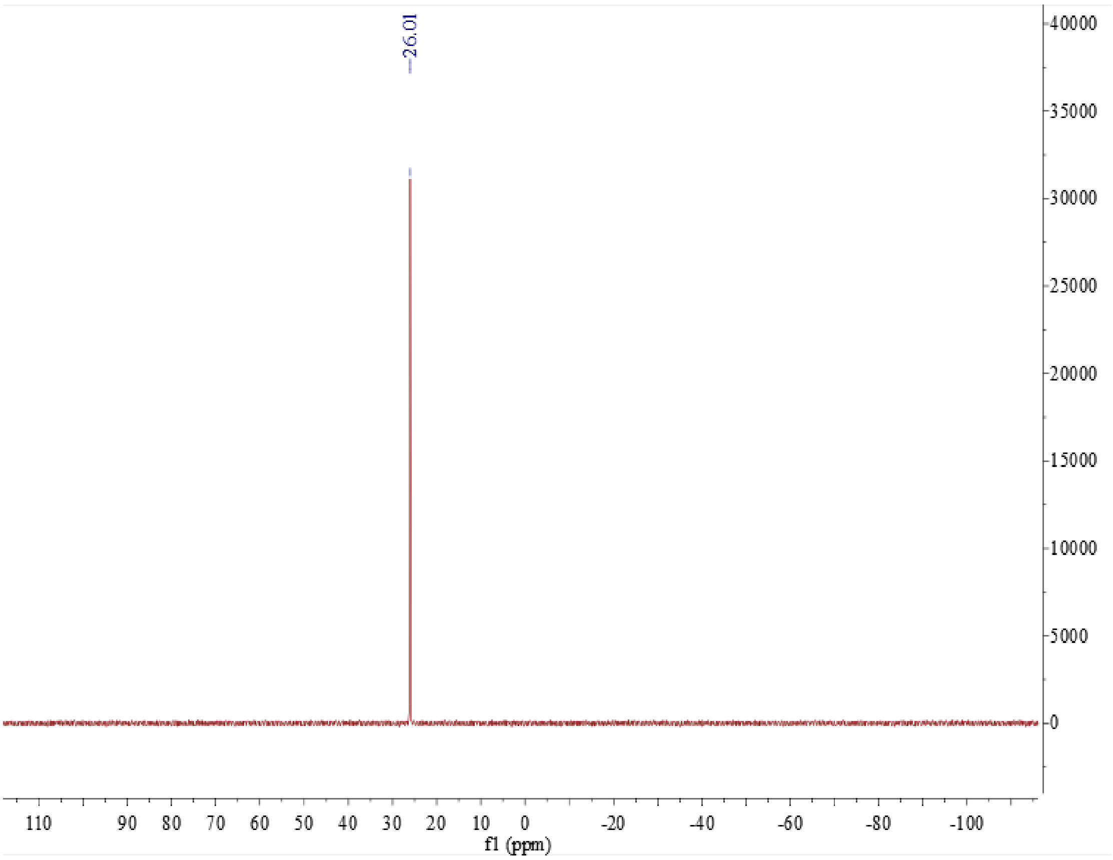

Supplementary Figure 16. ^{31}P NMR spectrum of **2a** in benzene-d_6 at 25 ºC

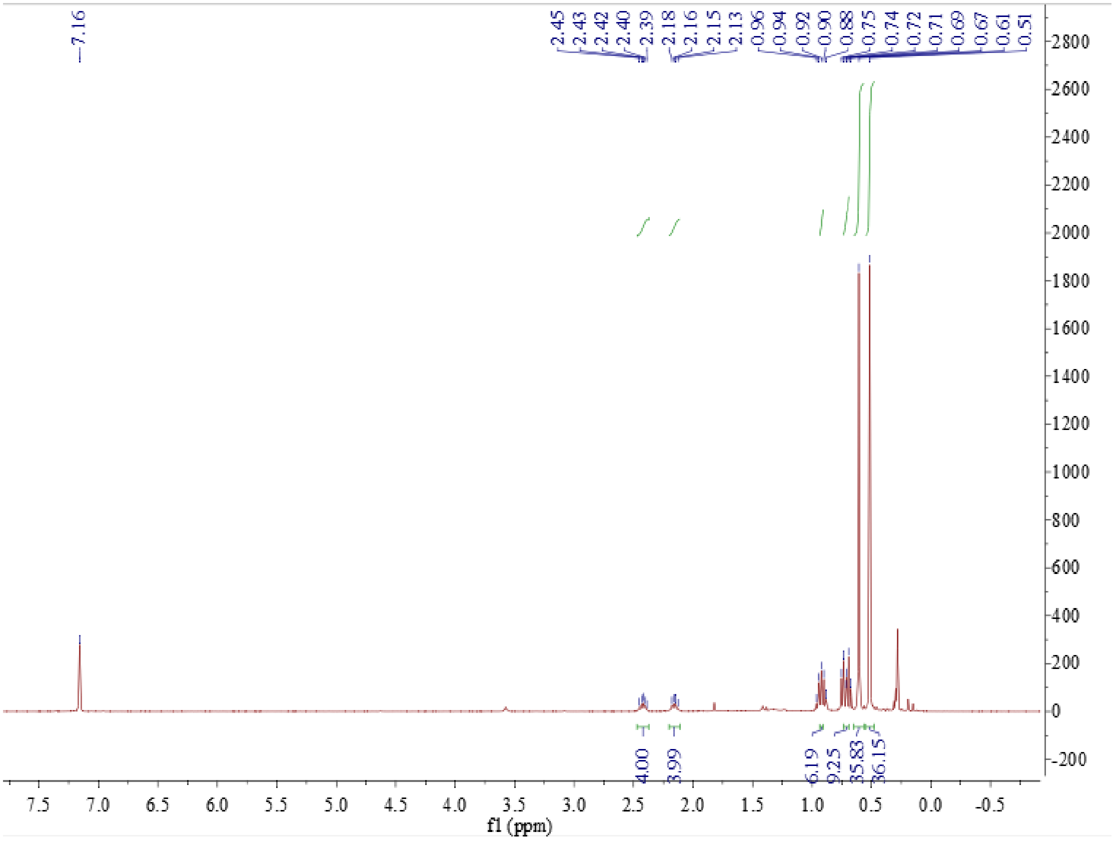

Supplementary Figure 17. ^{1}H NMR spectrum of **2b** in benzene-d_6 at 25 ºC

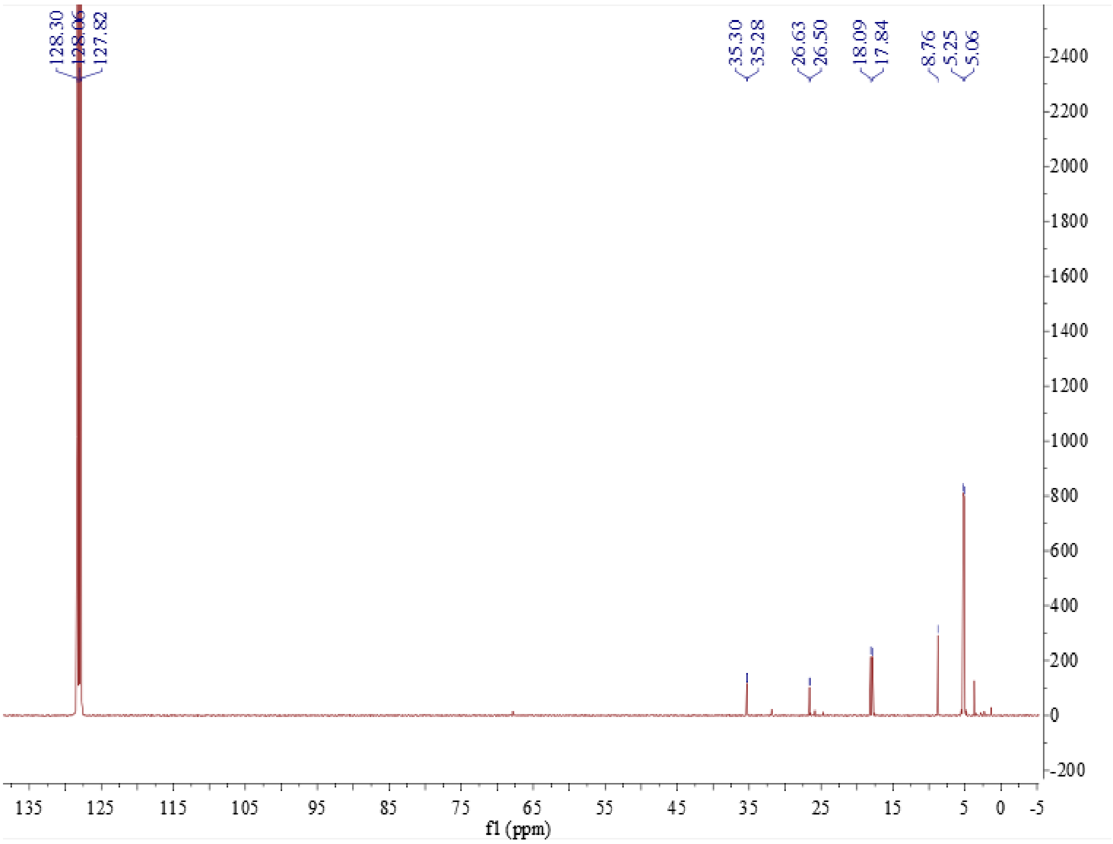

Supplementary Figure 18. ^{13}C NMR spectrum of **2b** in benzene-d_6 at 25 ºC

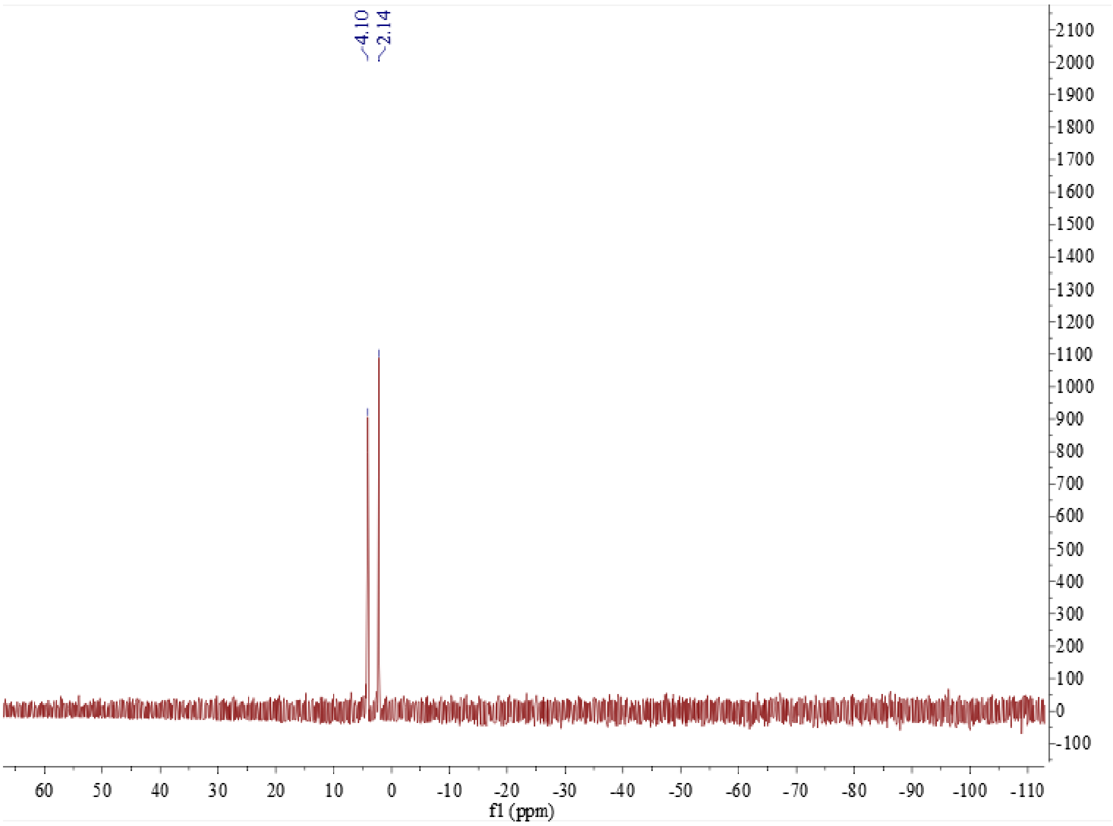

Supplementary Figure 19. ^{29}Si NMR spectrum of **2b** in benzene-d_6 at 25 ºC

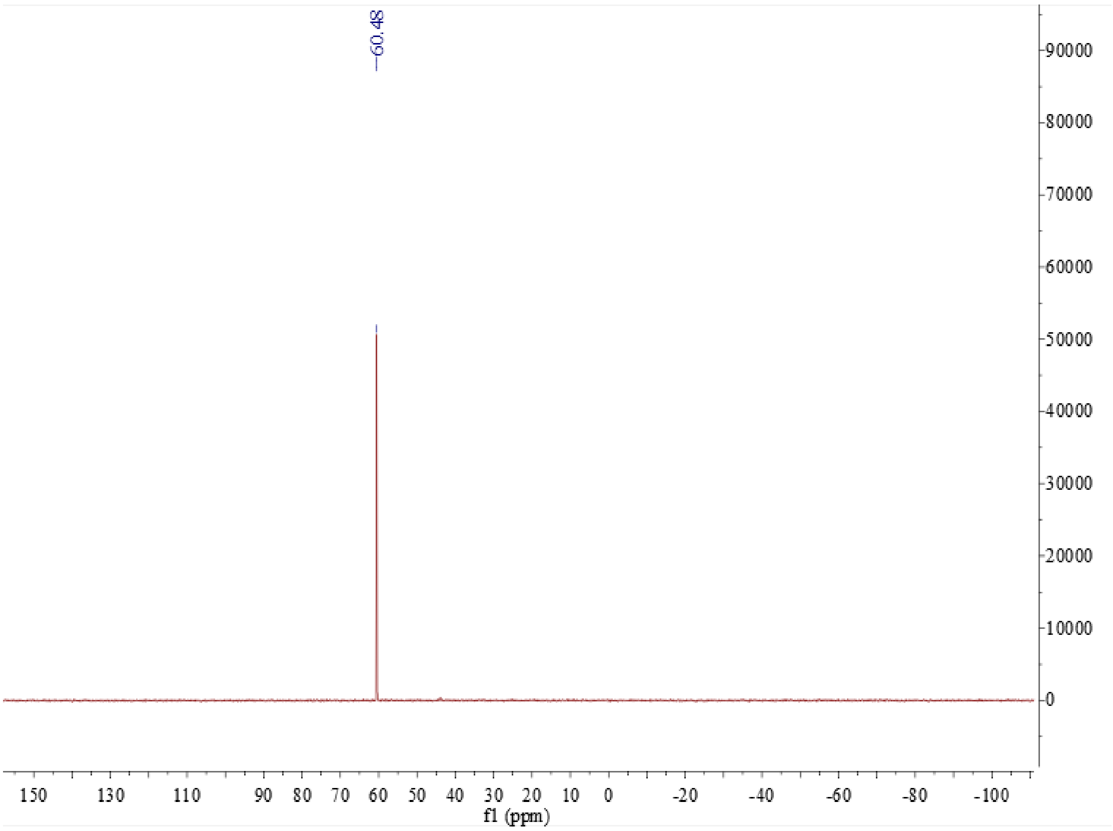

Supplementary Figure 20. ^{31}P NMR spectrum of **2b** in benzene-d_6 at 25 ºC

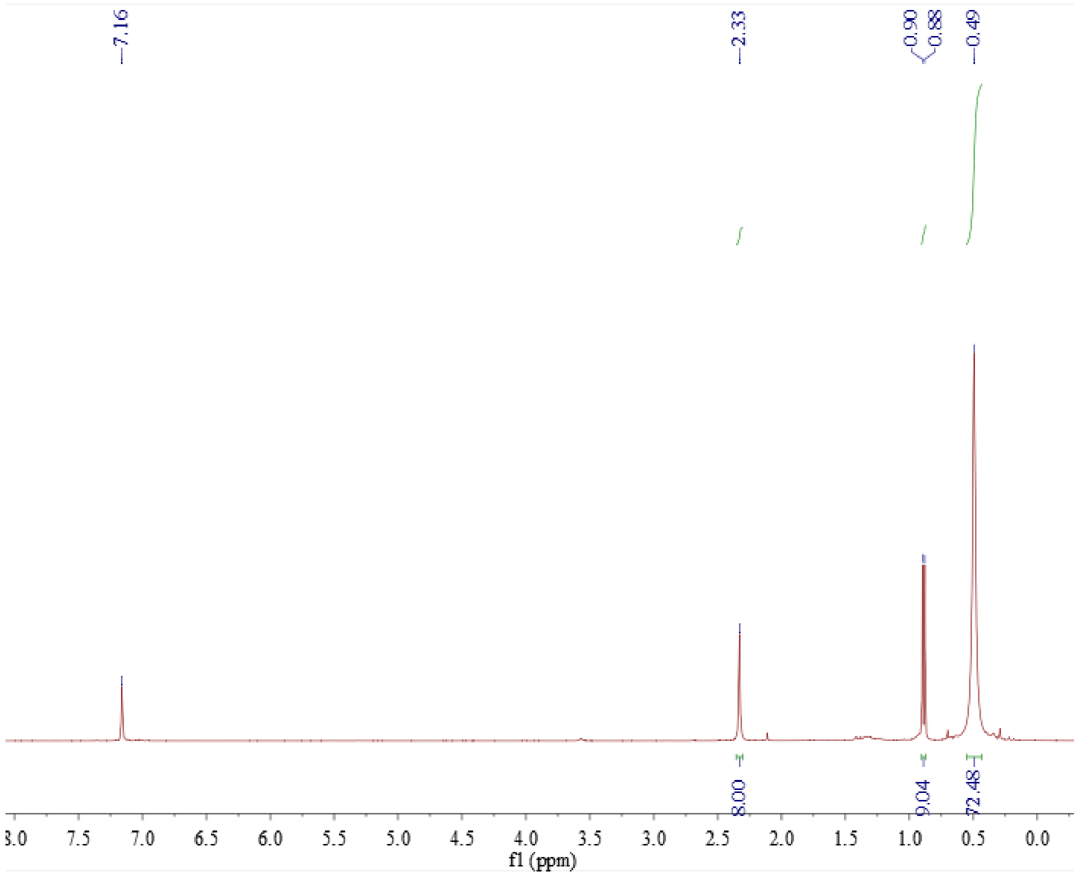

Supplementary Figure 21. ^{1}H NMR spectrum of **3a** in benzene-d_6 at 25 ºC

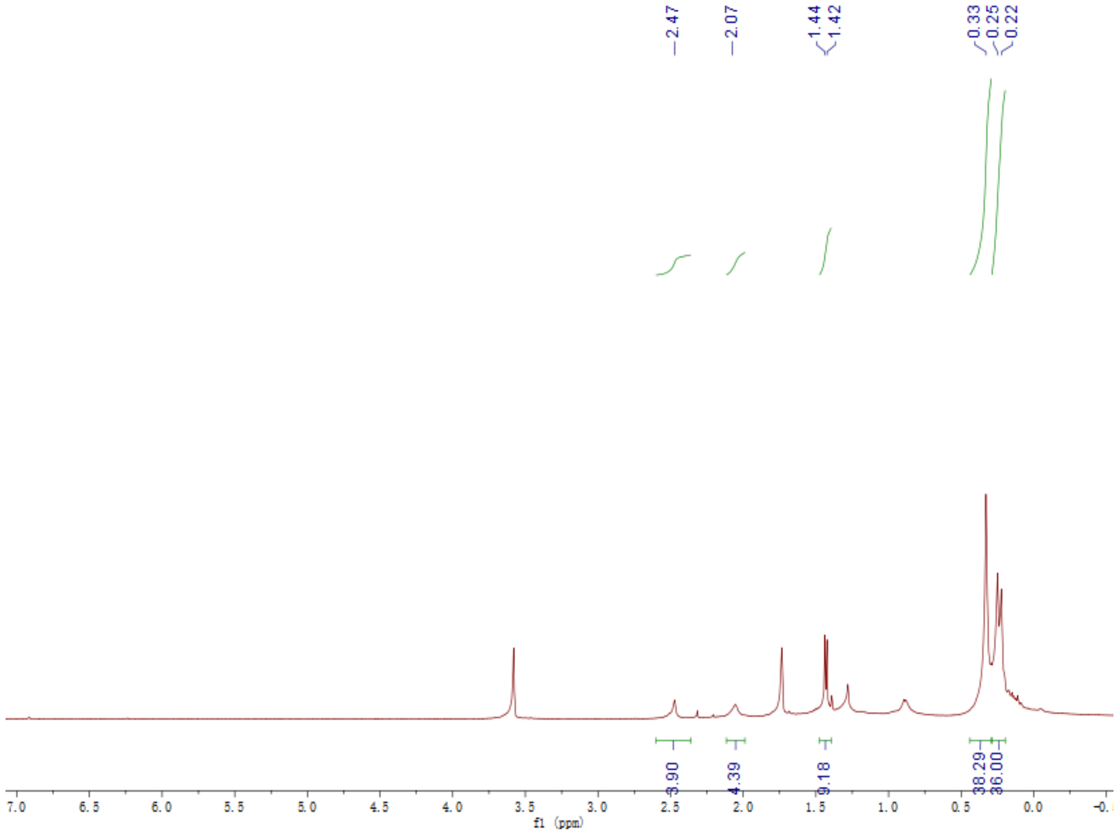

Supplementary Figure 22. ^{1}H NMR spectrum of **3a** in THF-d_8 at –30 ºC

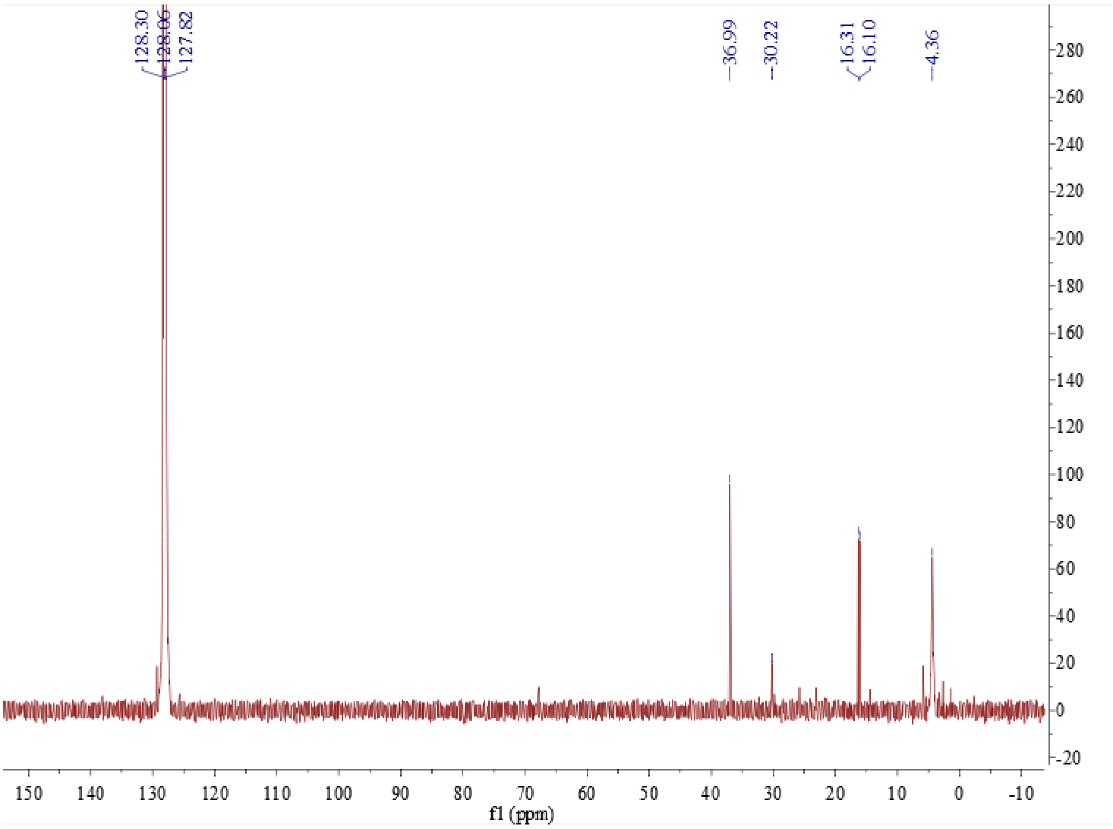

Supplementary Figure 23. ^{13}C NMR spectrum of **3a** in benzene-d_6 at 25 ºC

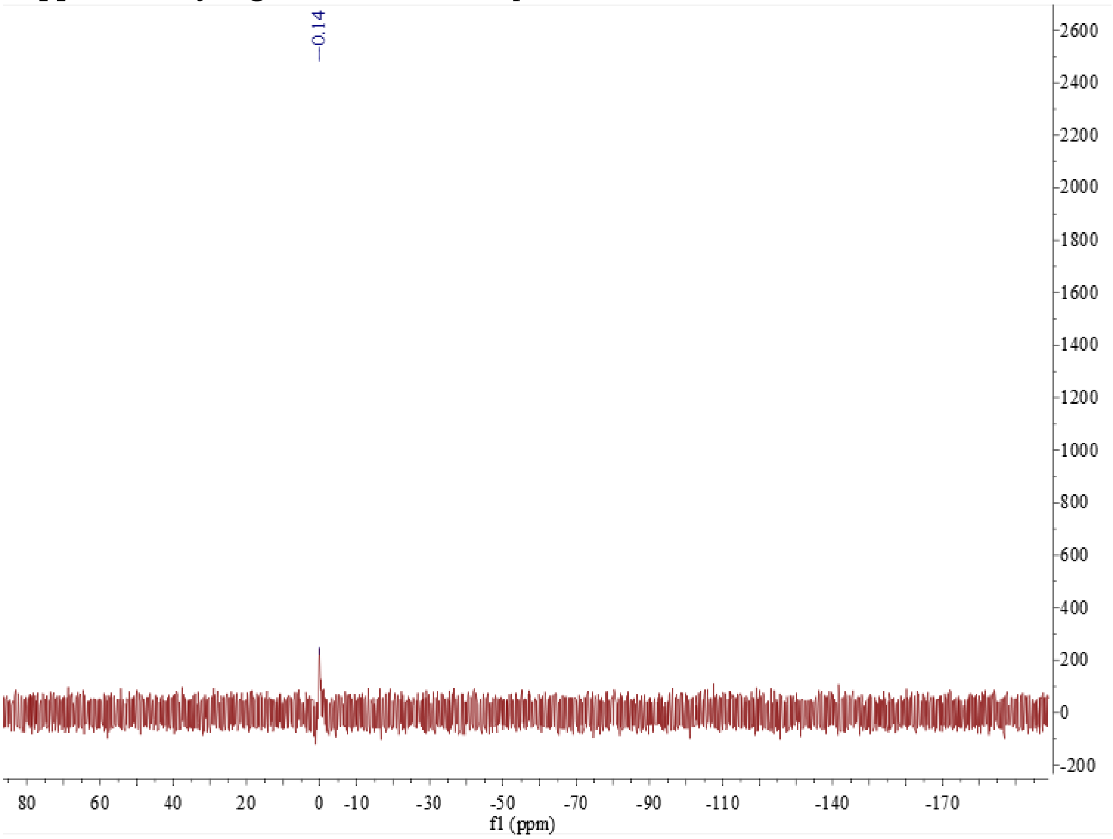

Supplementary Figure 24. ^{29}Si NMR spectrum of **3a** in benzene-d_6 at 25 ºC

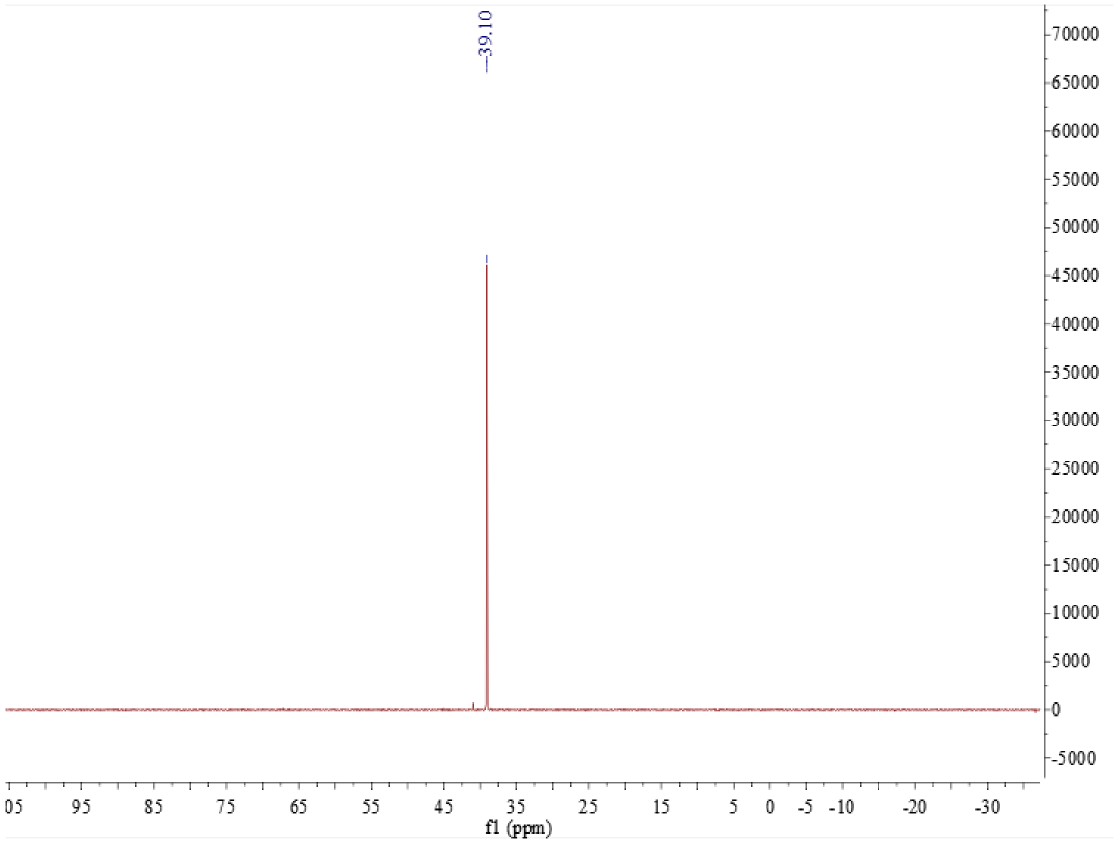

Supplementary Figure 25. ^{31}P NMR spectrum of **3a** in benzene-d_6 at 25 °C

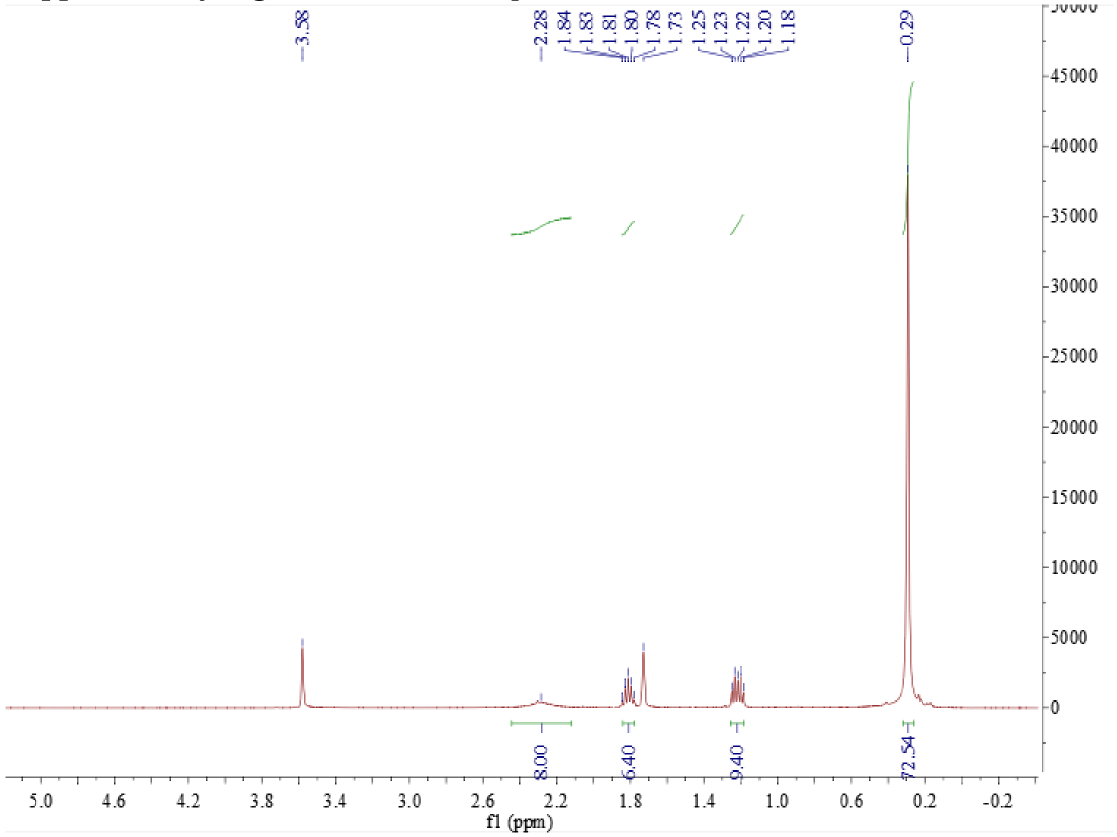

Supplementary Figure 26. ^{1}H NMR spectrum of **3b** in THF-d_8 at 25 °C

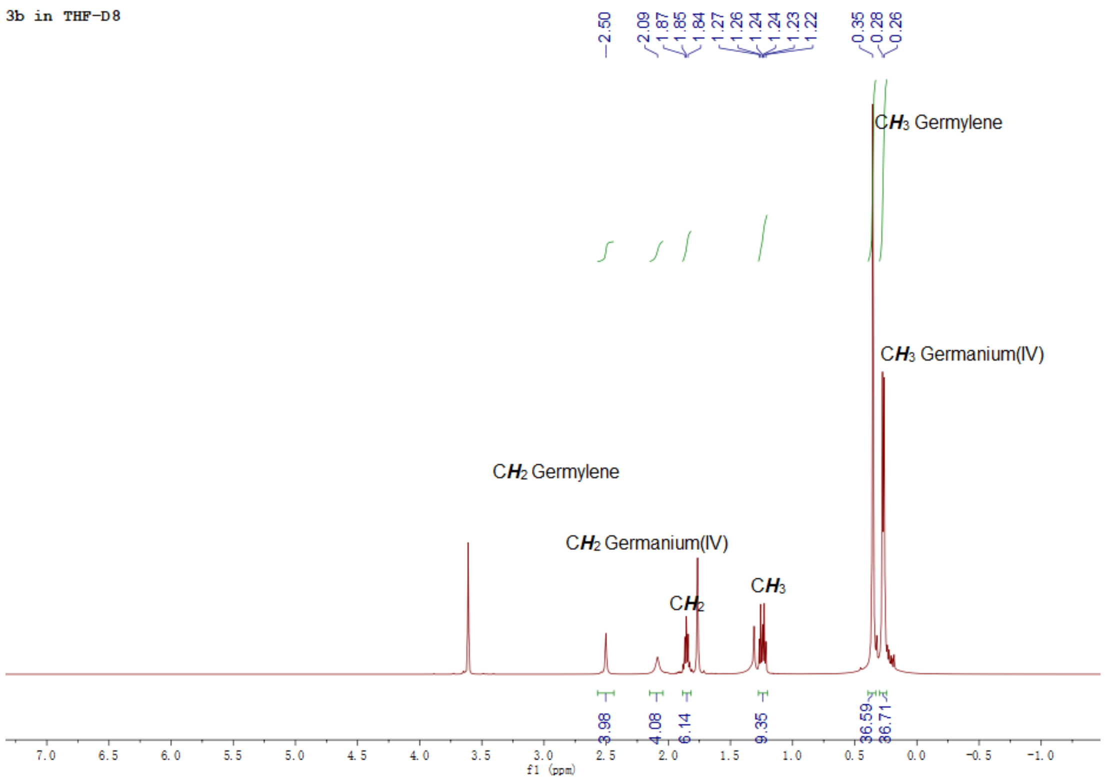

Supplementary Figure 27. ^{1}H NMR spectrum of **3b** in THF-d_8 at –30 °C

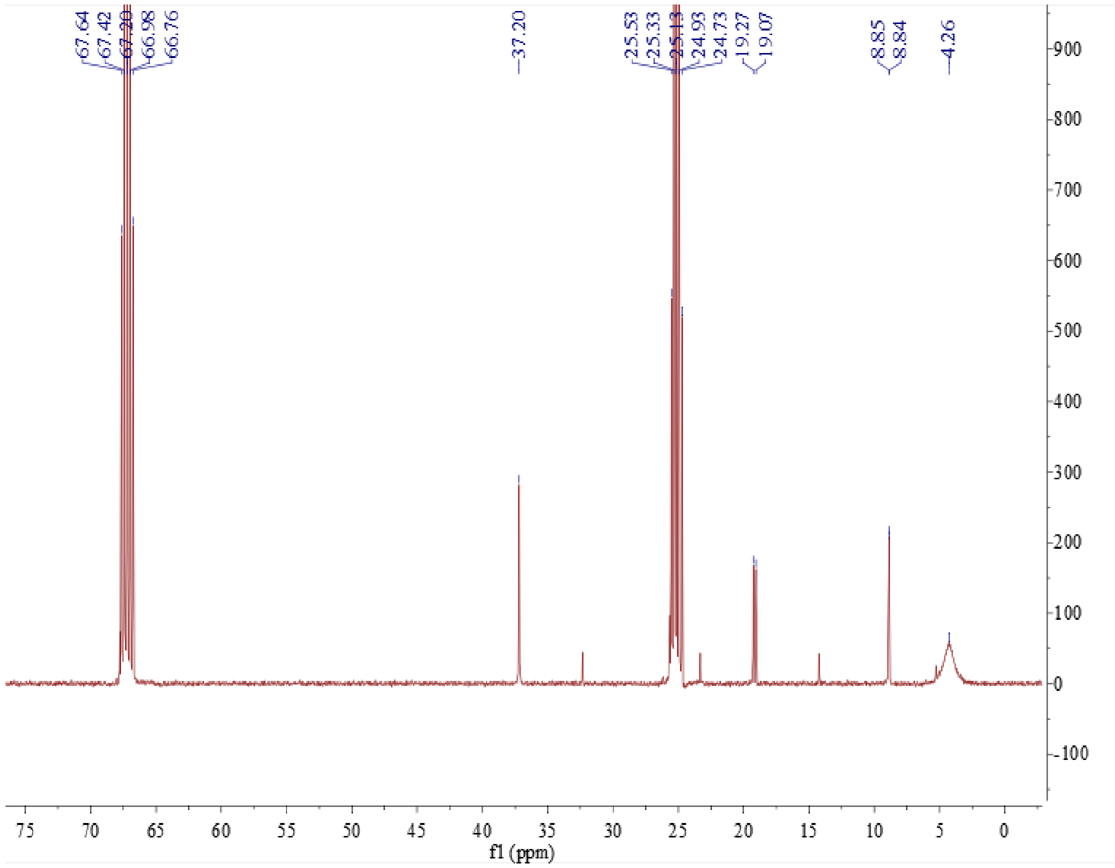

Supplementary Figure 28. ^{13}C NMR spectrum of **3b** in THF-d_8 at 25 °C

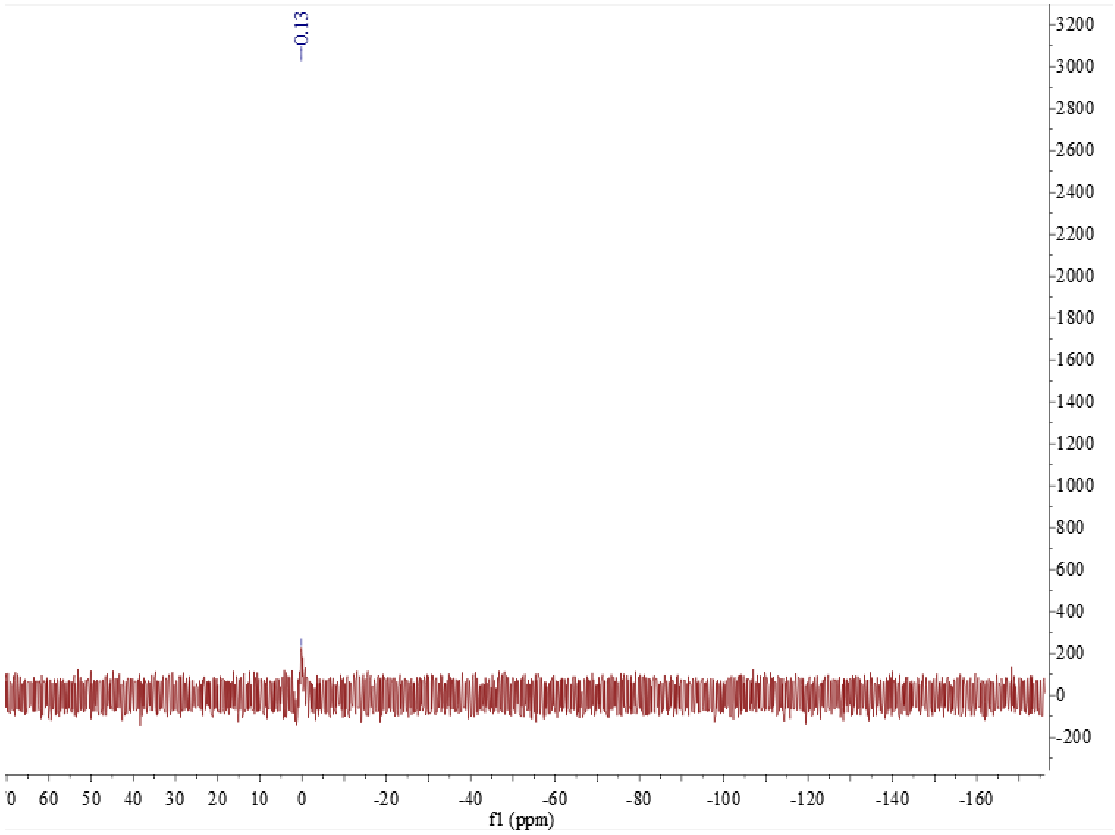

Supplementary Figure 29. ^{29}Si NMR spectrum of **3b** in THF-d_8 at 25 ºC

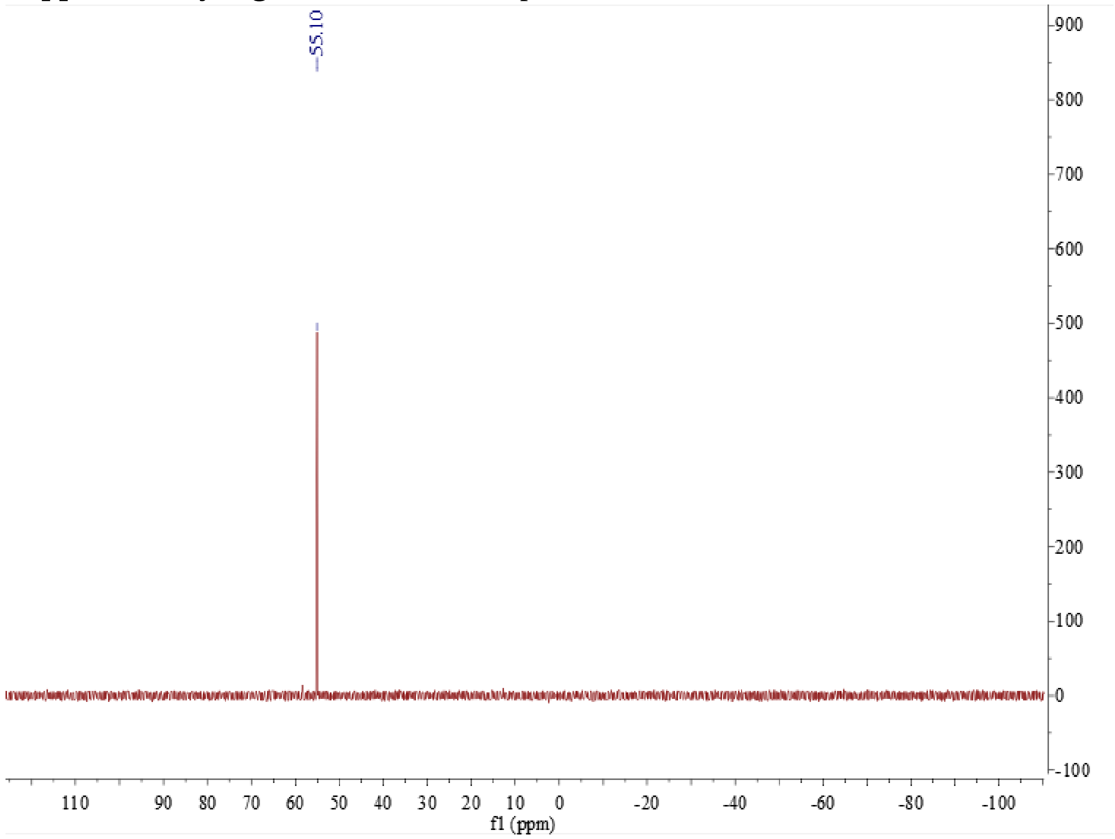

Supplementary Figure 30. ^{31}P NMR spectrum of **3b** in THF-d_8 at 25 ºC

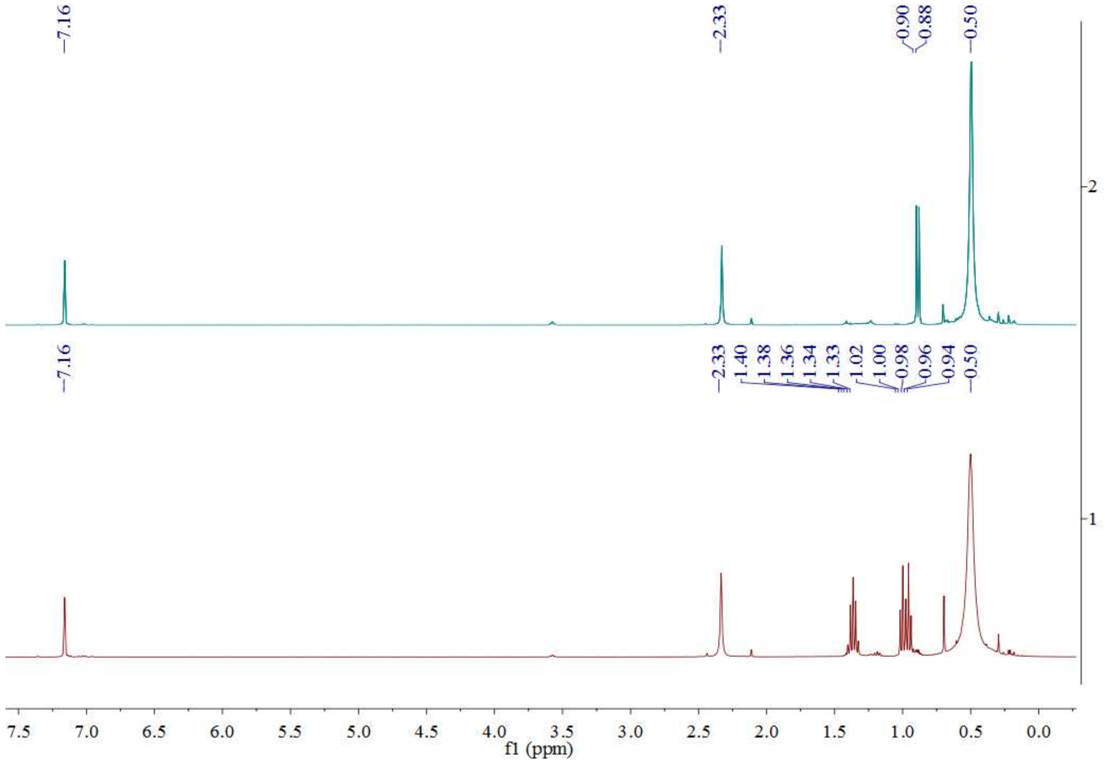

Supplementary Figure 31. ^{1}H NMR spectra of **3a** and **3b** in C_6D_6 at 25 ºC

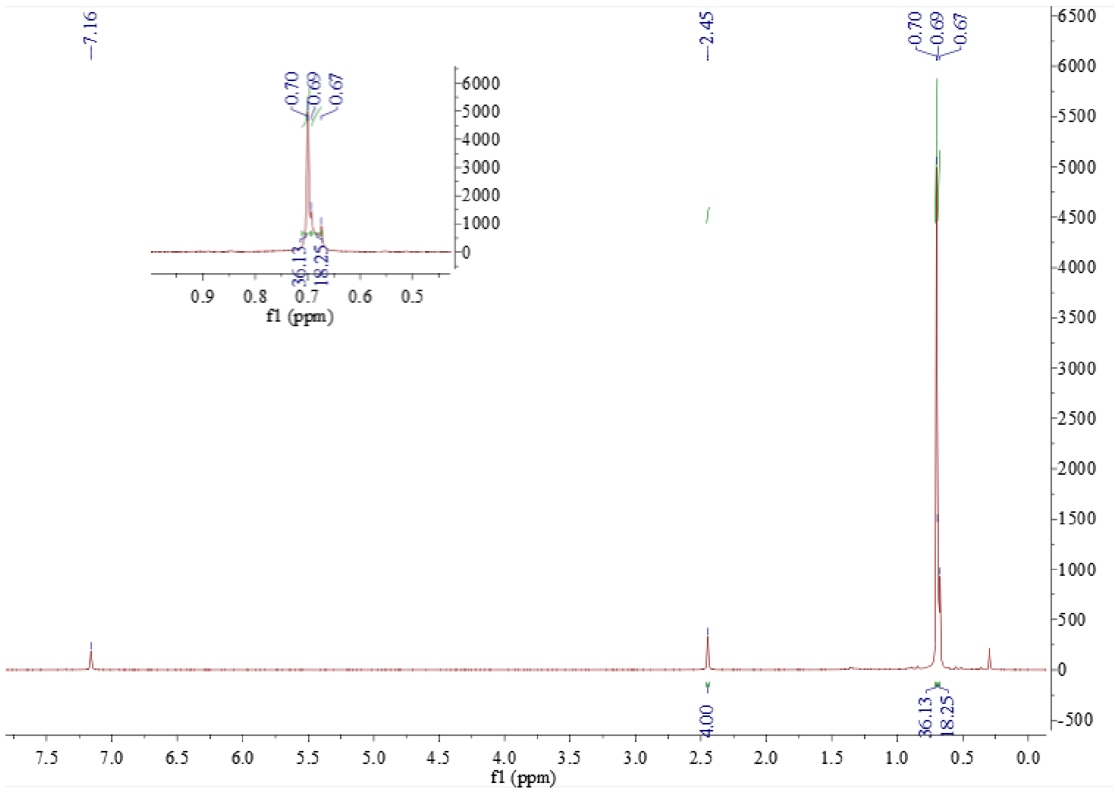

Supplementary Figure 32. ^{1}H NMR spectrum of **4** in C_6D_6 at 25 ºC

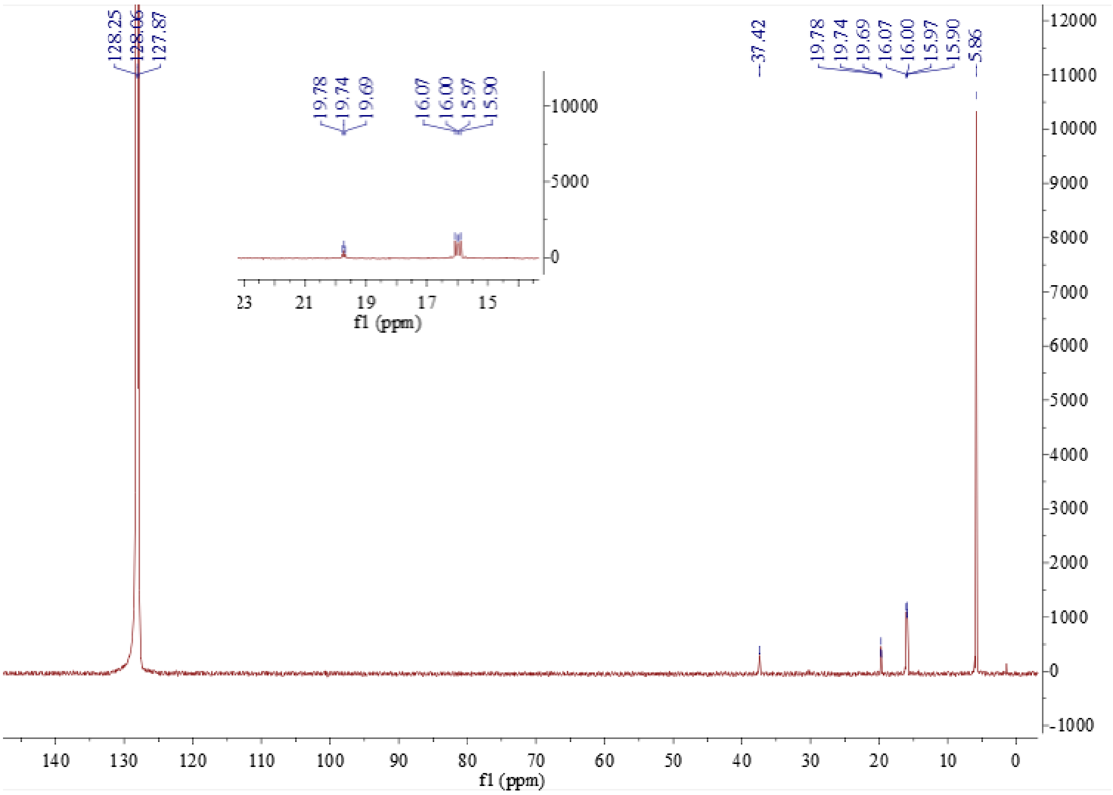

Supplementary Figure 33. ^{13}C NMR spectrum of **4** in C_6D_6 at 25 ºC

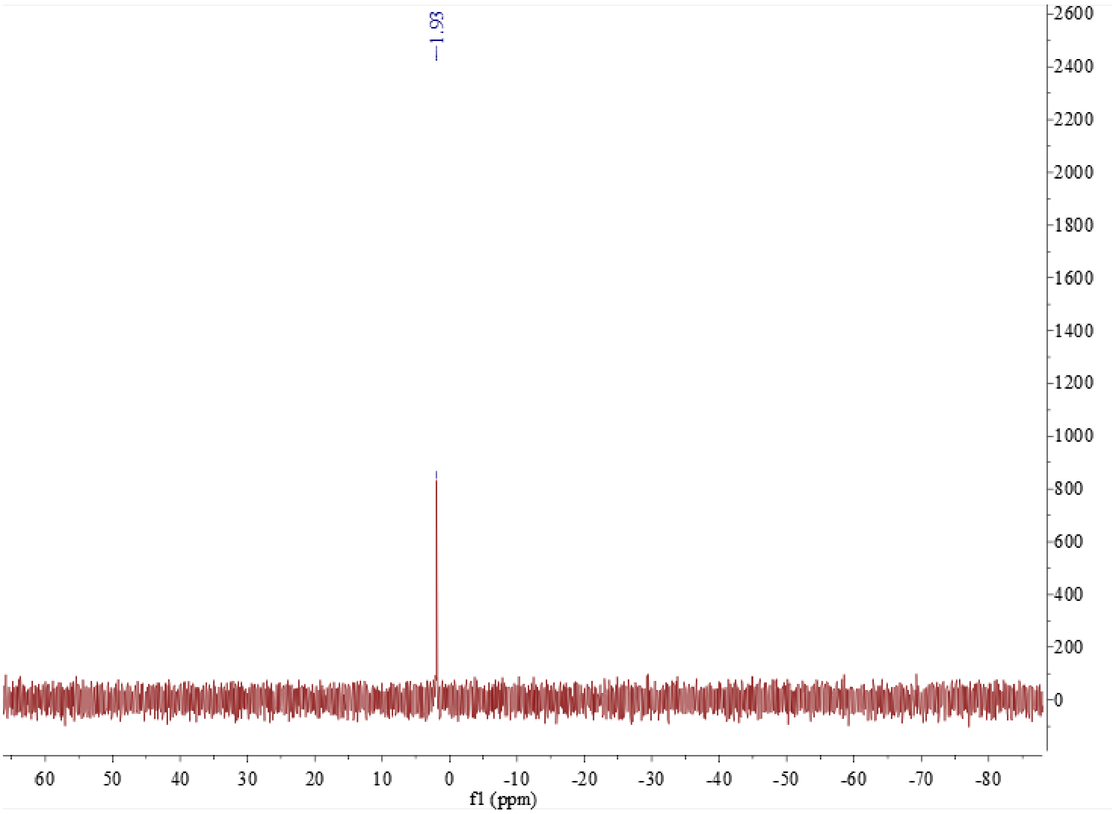

Supplementary Figure 34. ^{29}Si NMR spectrum of **4** in C_6D_6 at 25 ºC

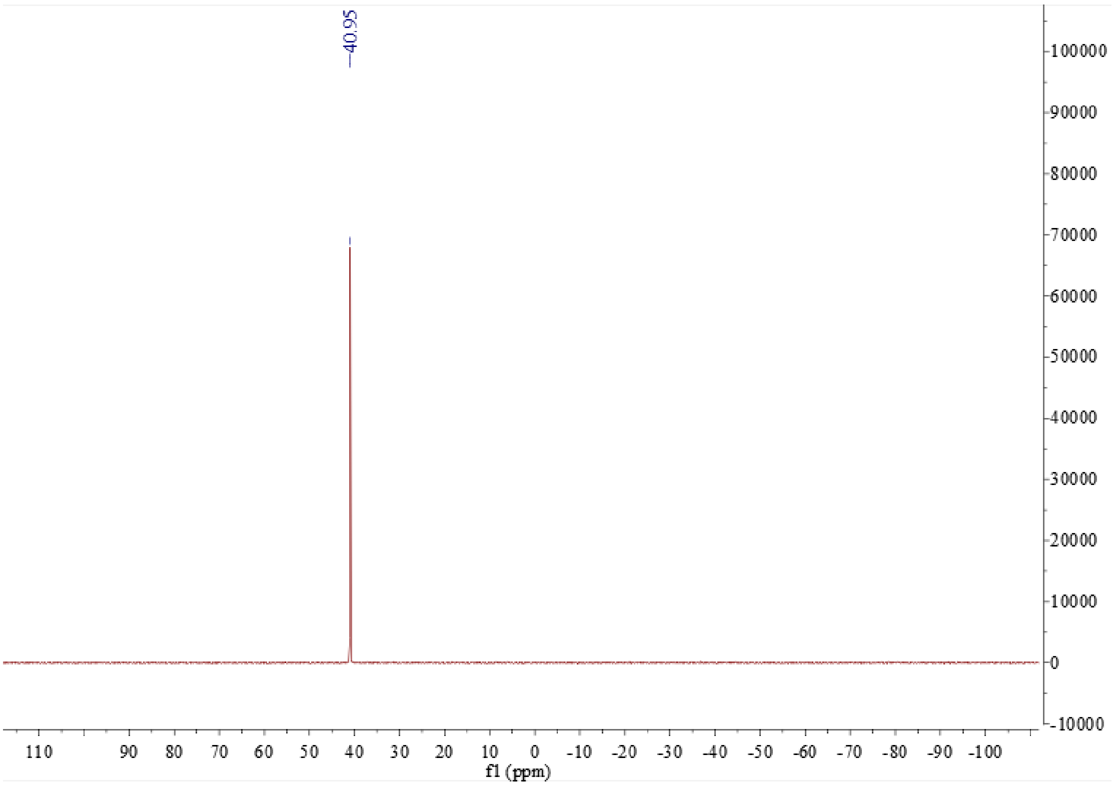

Supplementary Figure 35. ^{31}P NMR spectrum of **4** in C_6D_6 at 25 ºC

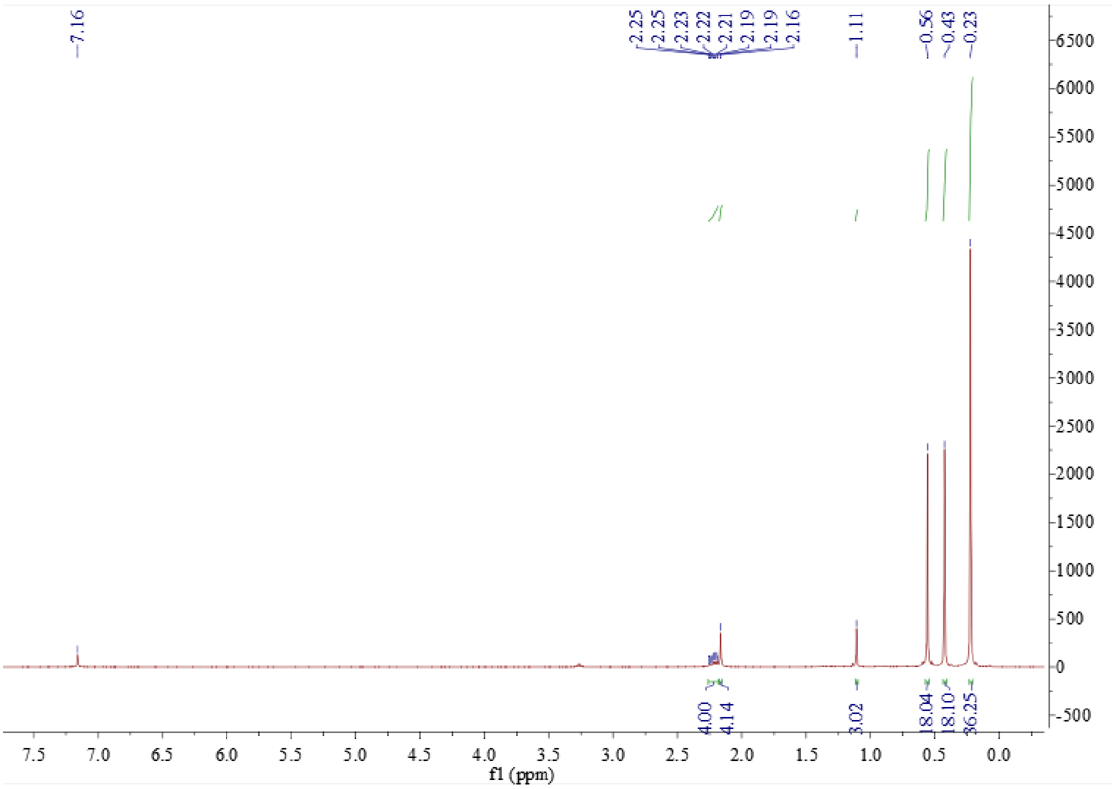

Supplementary Figure 36. ^{1}H NMR spectrum of **5** in C_6D_6 at 25 ºC

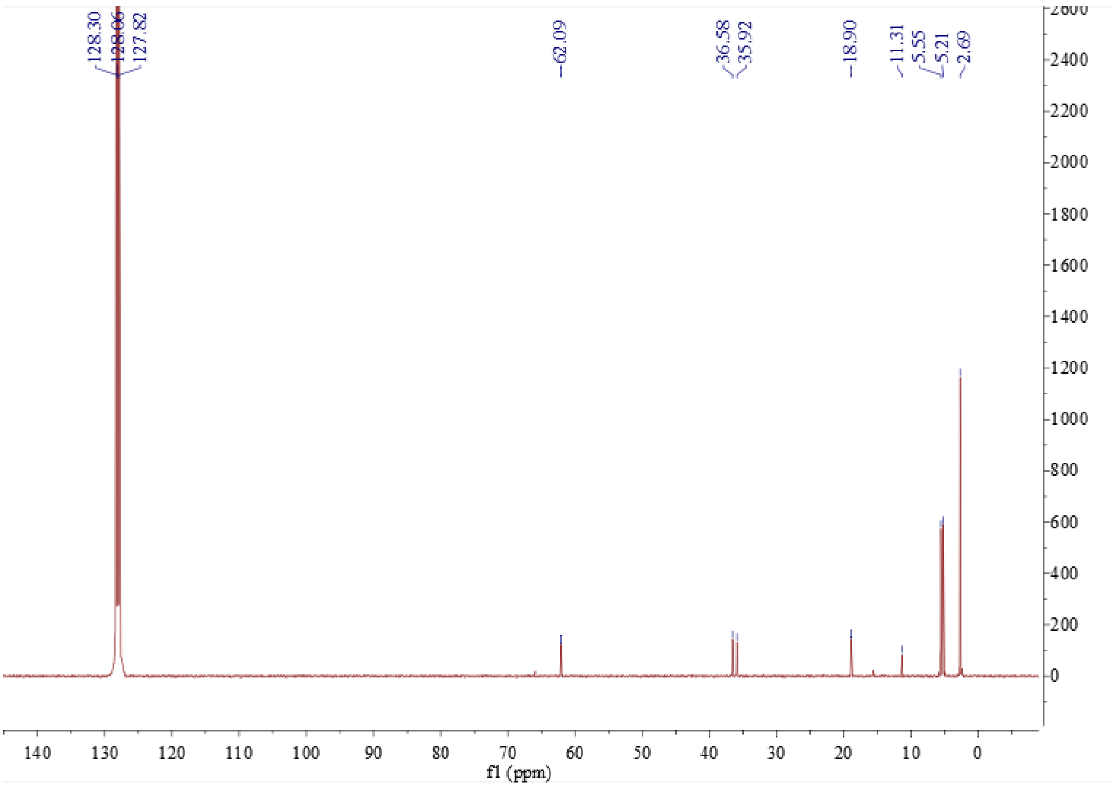

Supplementary Figure 37. ^{13}C NMR spectrum of **5** in C_6D_6 at 25 ºC

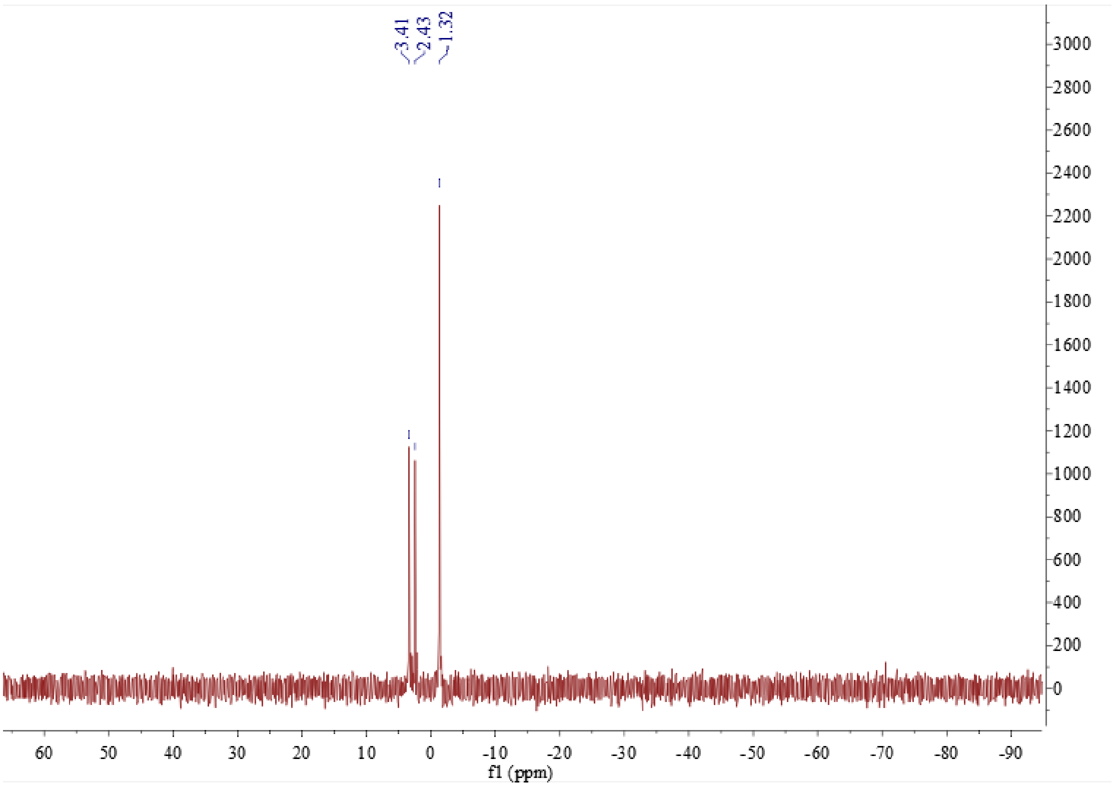

Supplementary Figure 38. ^{29}Si NMR spectrum of **5** in C_6D_6 at 25 ºC

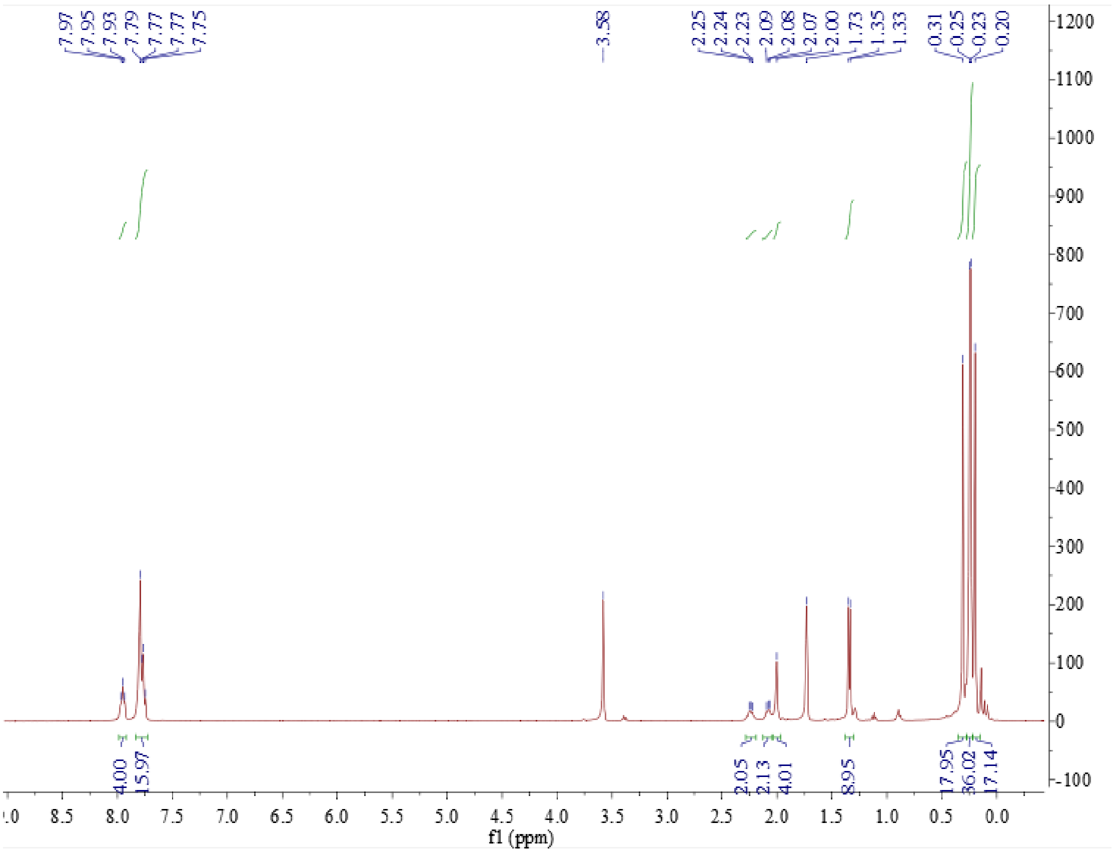

Supplementary Figure 39. ^{1}H NMR spectrum of **7** in THF-d_8 at 25 ºC

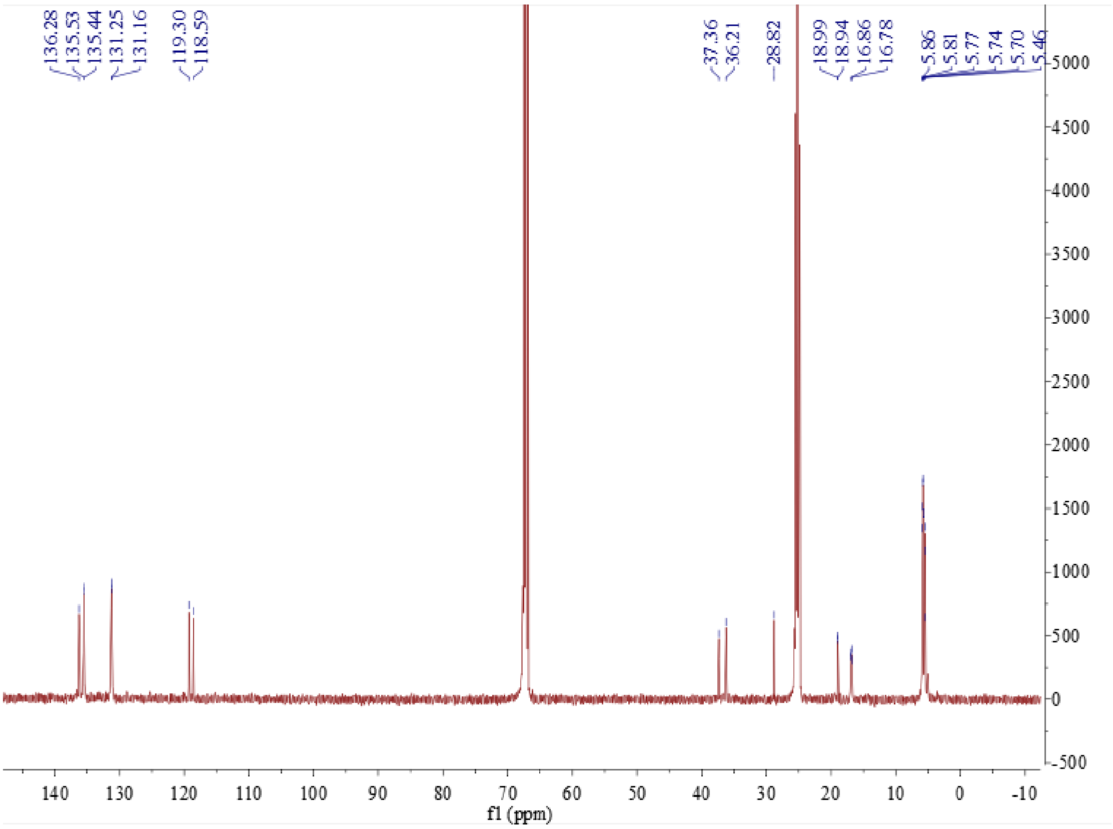

Supplementary Figure 40. ^{13}C NMR spectrum of **7** in THF-d_8 at 25 ºC

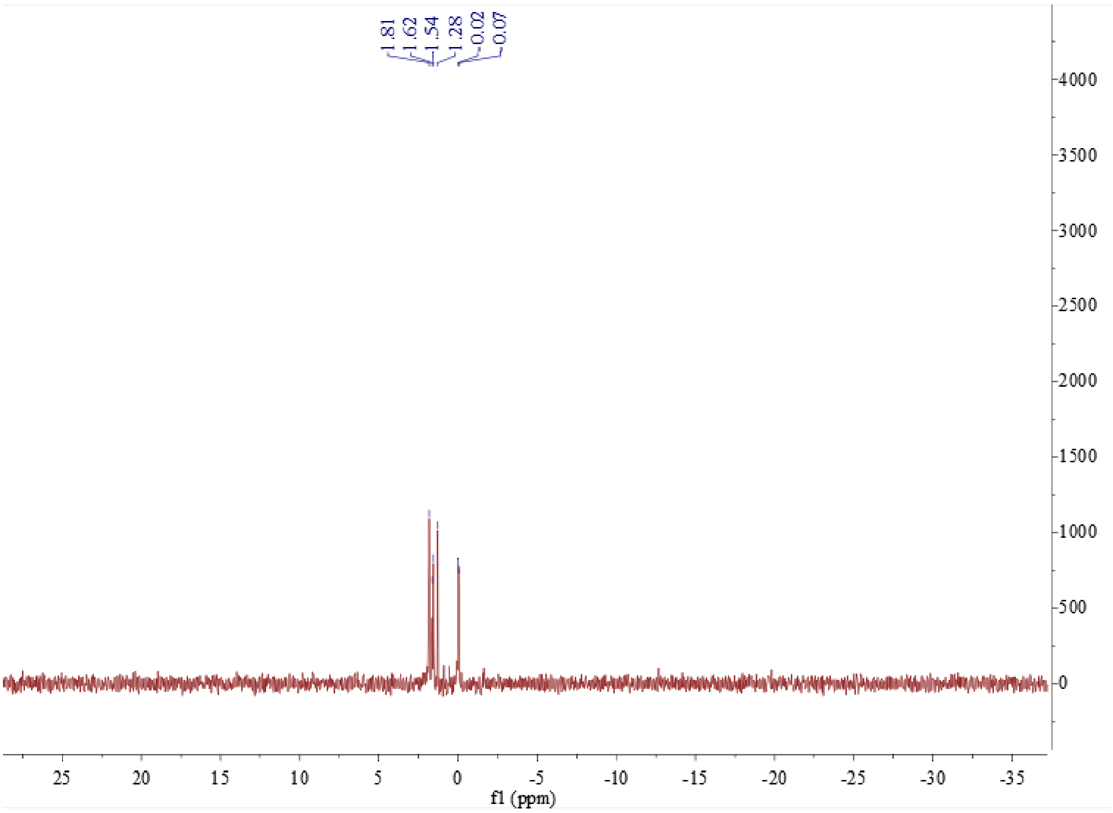

Supplementary Figure 41. ^{29}Si NMR spectrum of **7** in THF-d_8 at 25 ºC

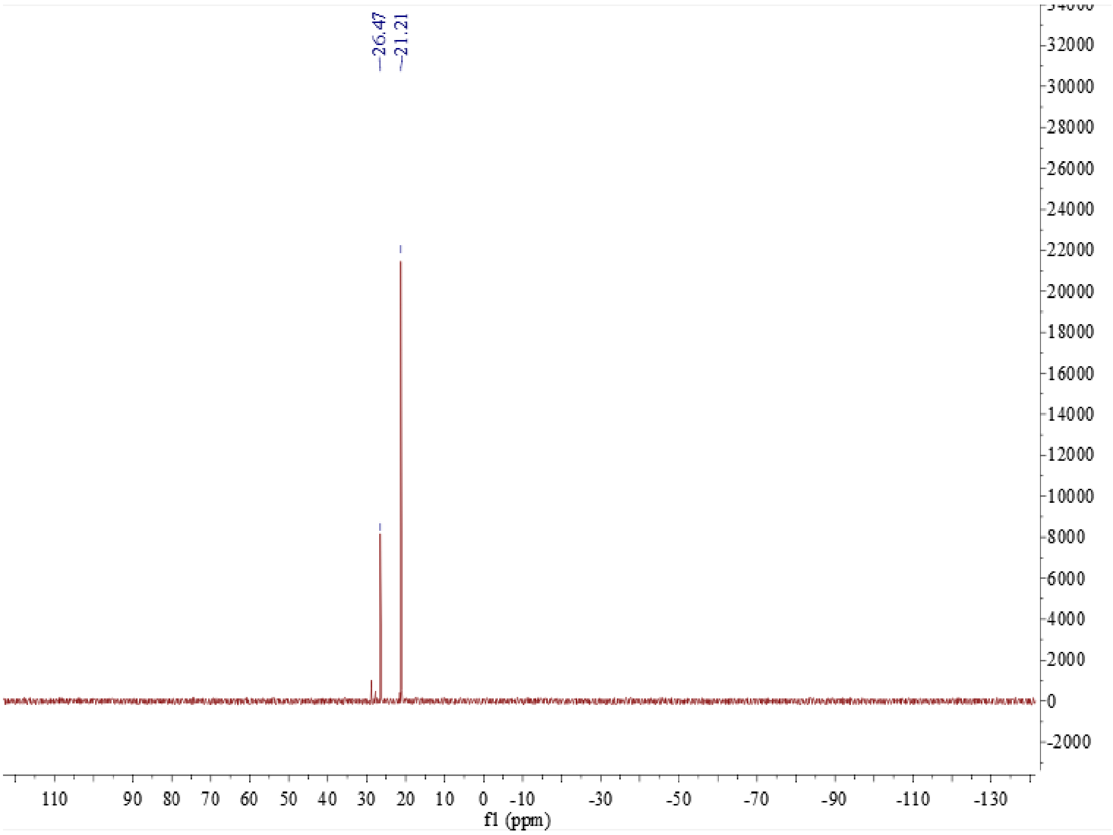

Supplementary Figure 42. ^{31}P NMR spectrum of **7** in THF-d_8 at 25 ºC

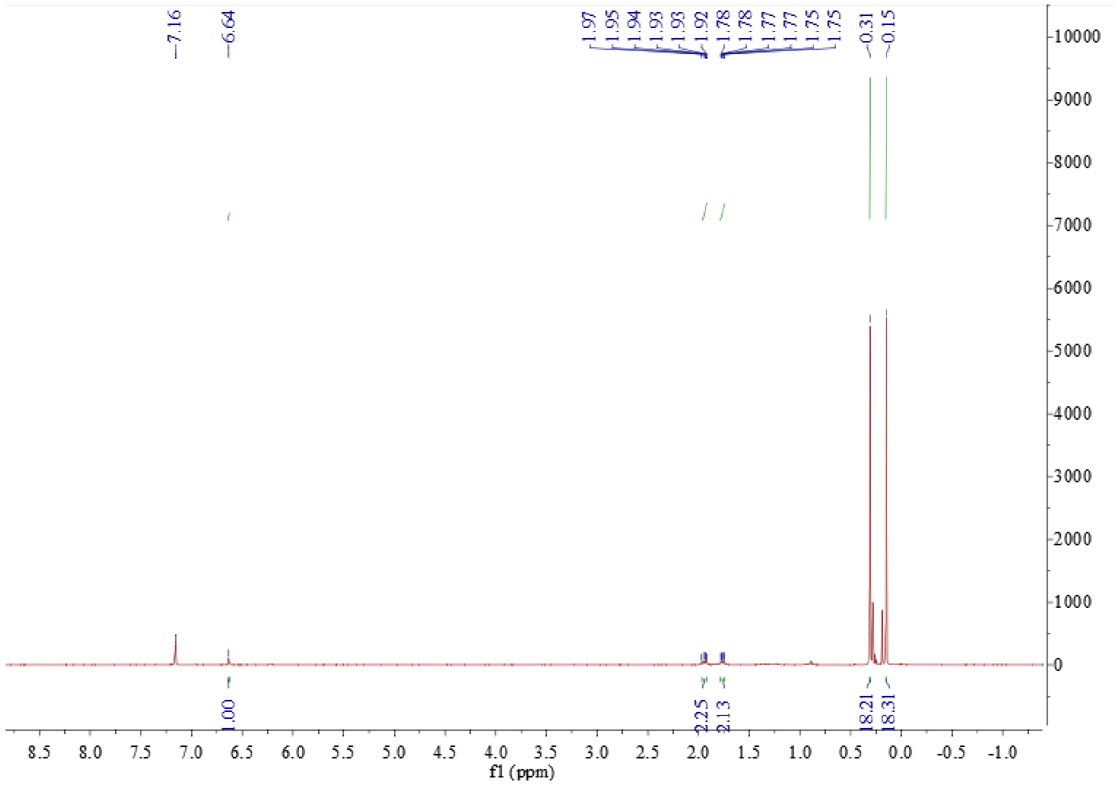

Supplementary Figure 43. ^{1}H NMR spectrum of **1•HCl** in C_6D_6 at 25 ºC

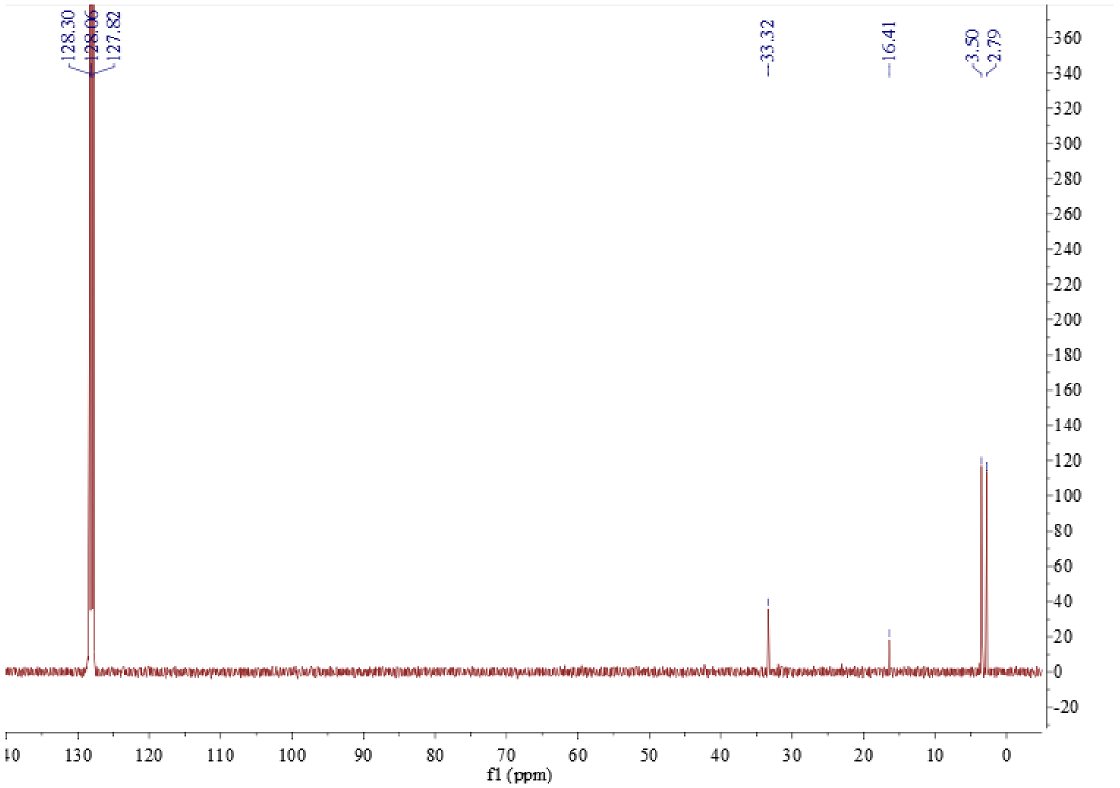

Supplementary Figure 44. ^{13}C NMR spectrum of **1•HCl** in C_6D_6 at 25 ºC

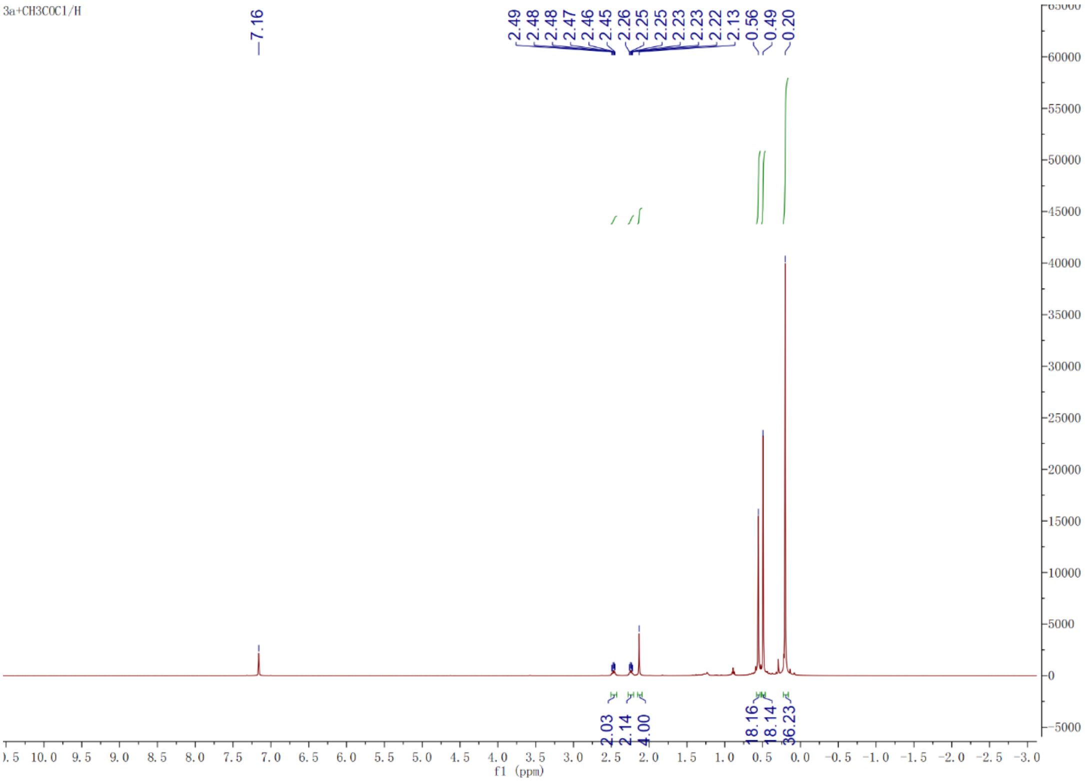

Supplementary Figure 45. ^{1}H NMR spectrum of **9** in C_6D_6 at 25 ºC

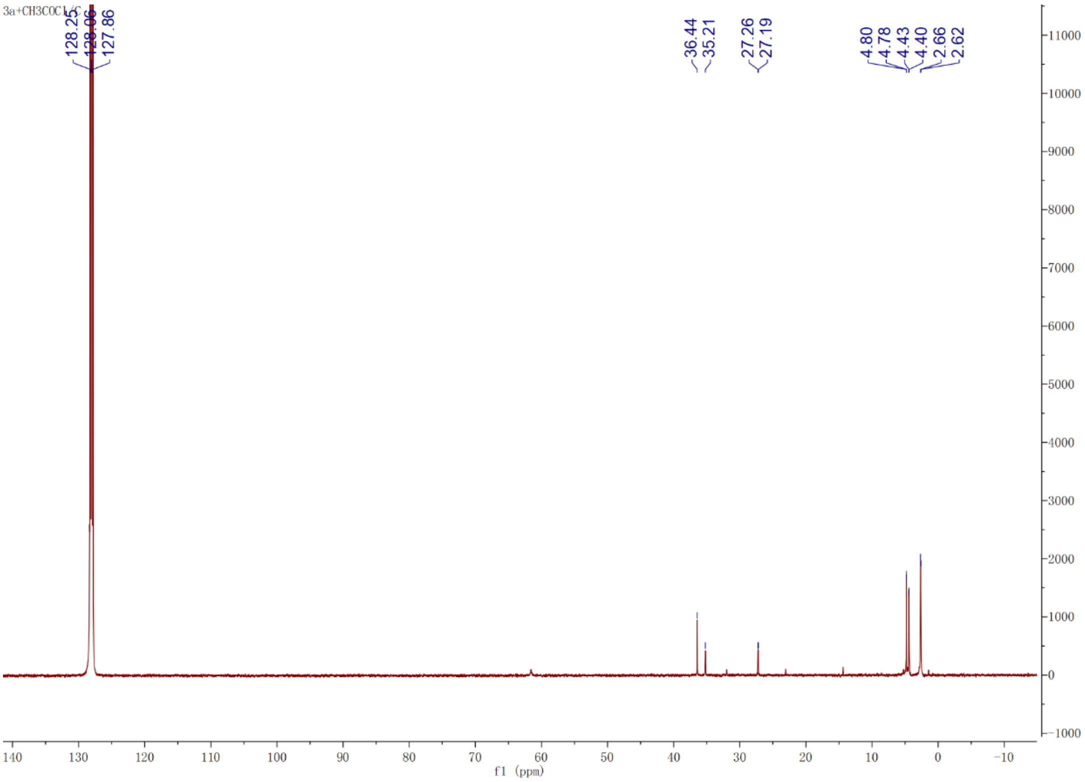

Supplementary Figure 46. ^{13}C NMR spectrum of **9** in C_6D_6 at 25 ºC

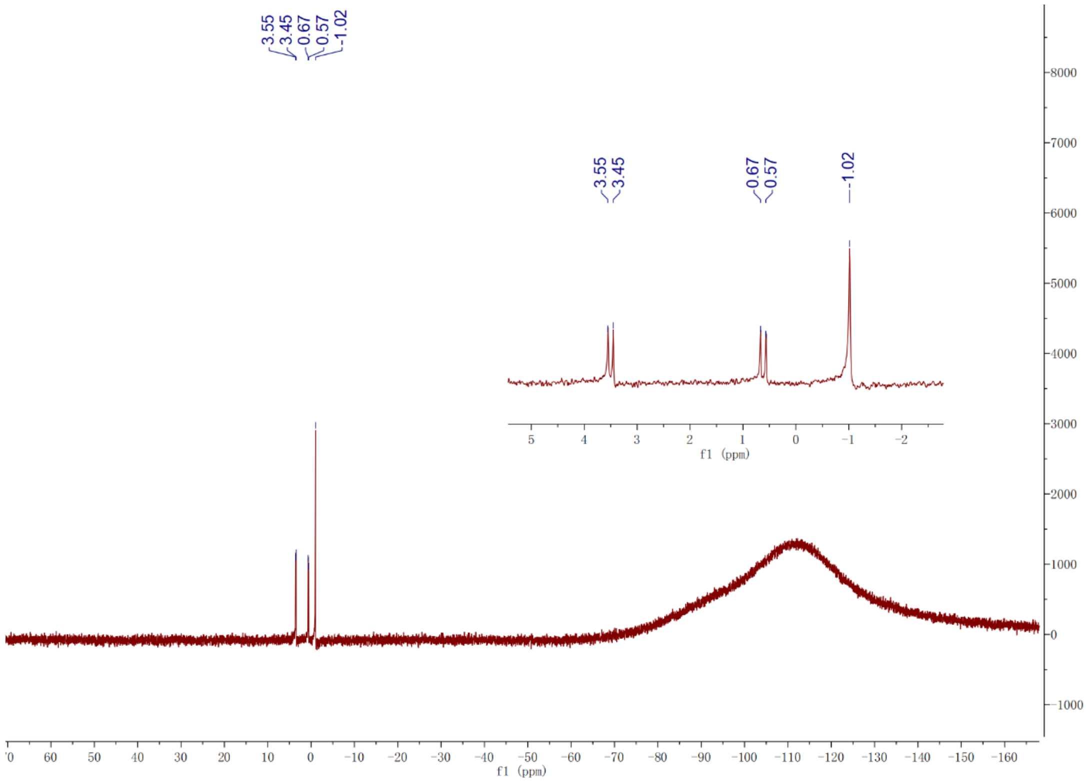

Supplementary Figure 47. ^{29}Si NMR spectrum of **9** in C_6D_6 at 25 ºC

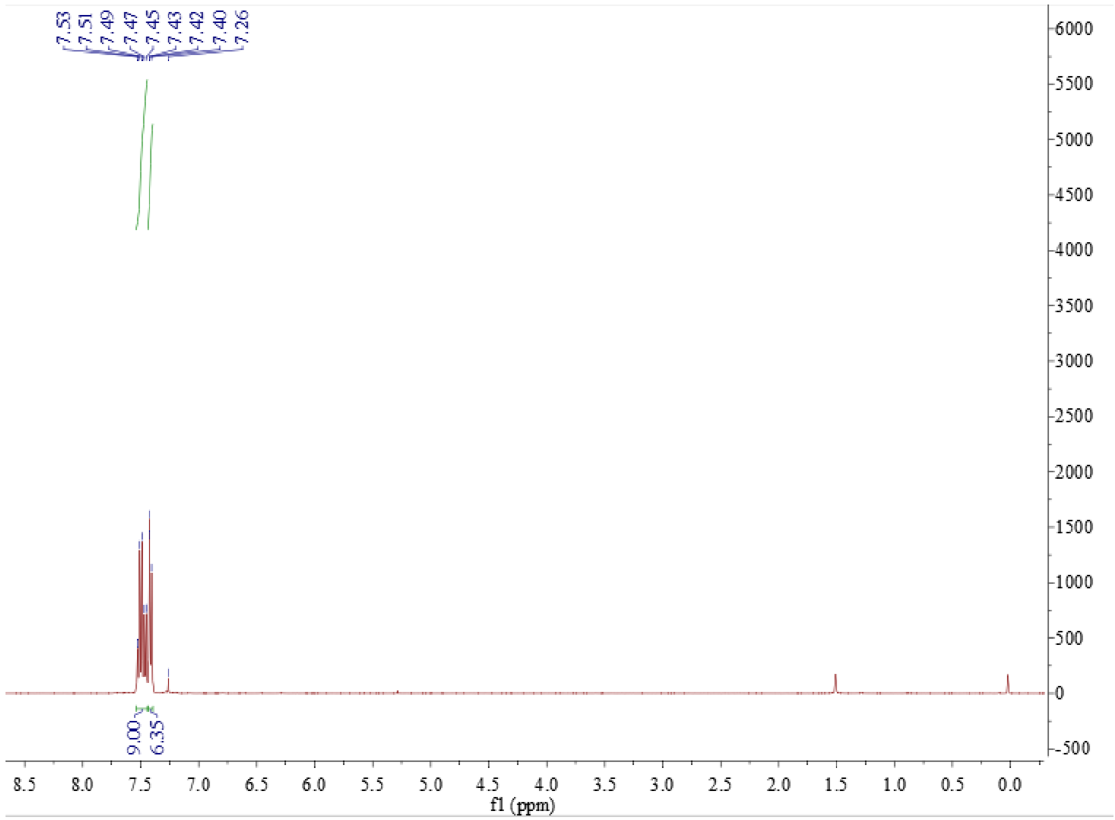

Supplementary Figure 48. ^{1}H NMR spectrum of **11a** in $CDCl_3$ at 25 ºC

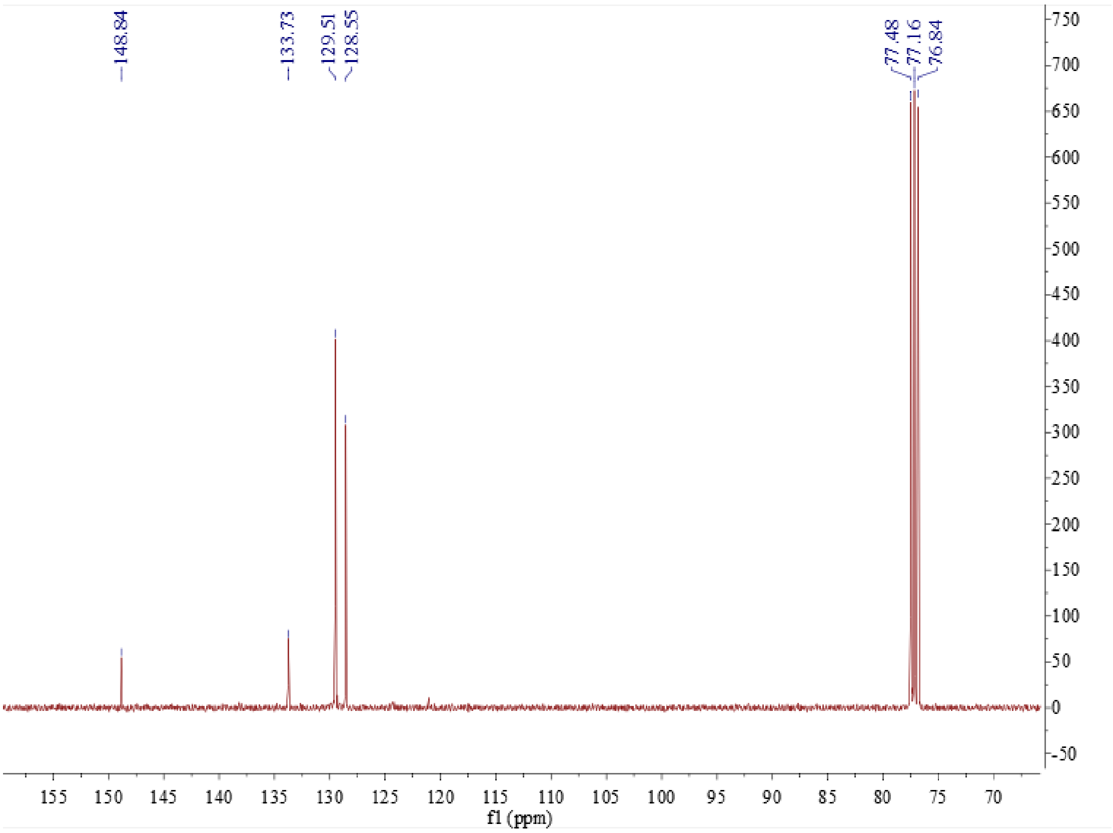

Supplementary Figure 49. ^{13}C NMR spectrum of **11a** in $CDCl_3$ at 25 ºC

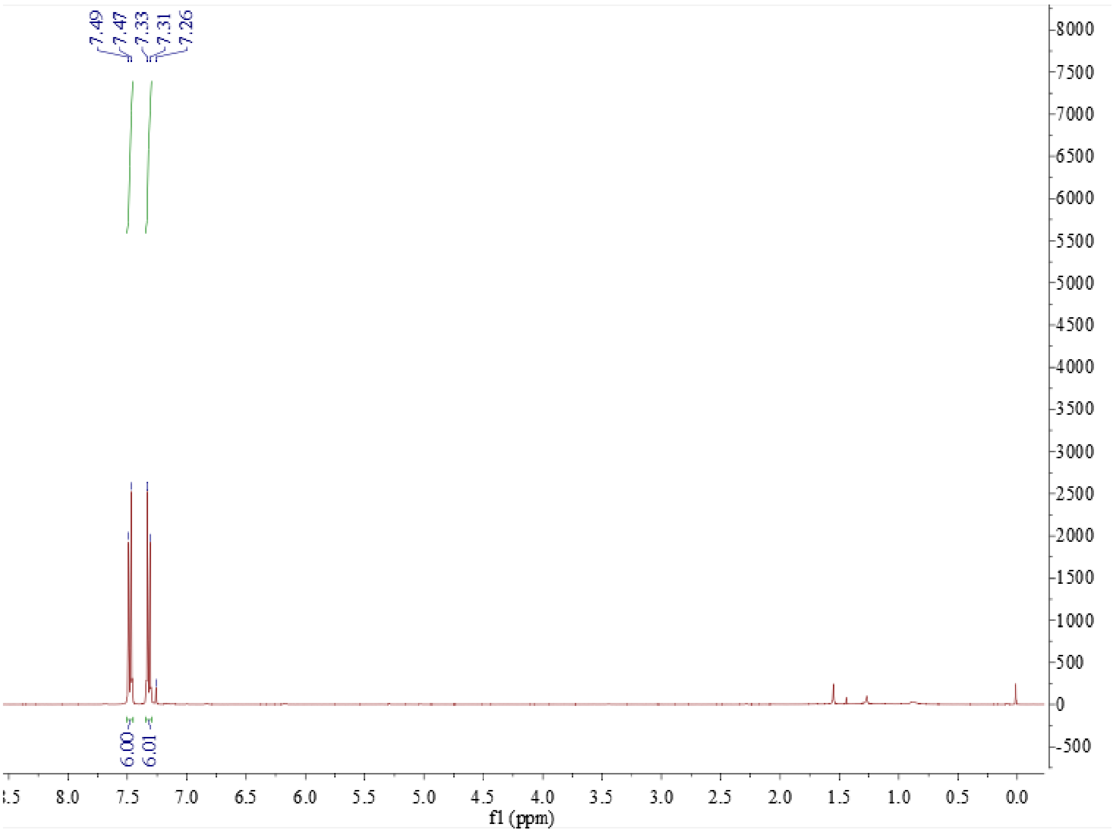

Supplementary Figure 50. ^{1}H NMR spectrum of **11b** in $CDCl_3$ at 25 ºC

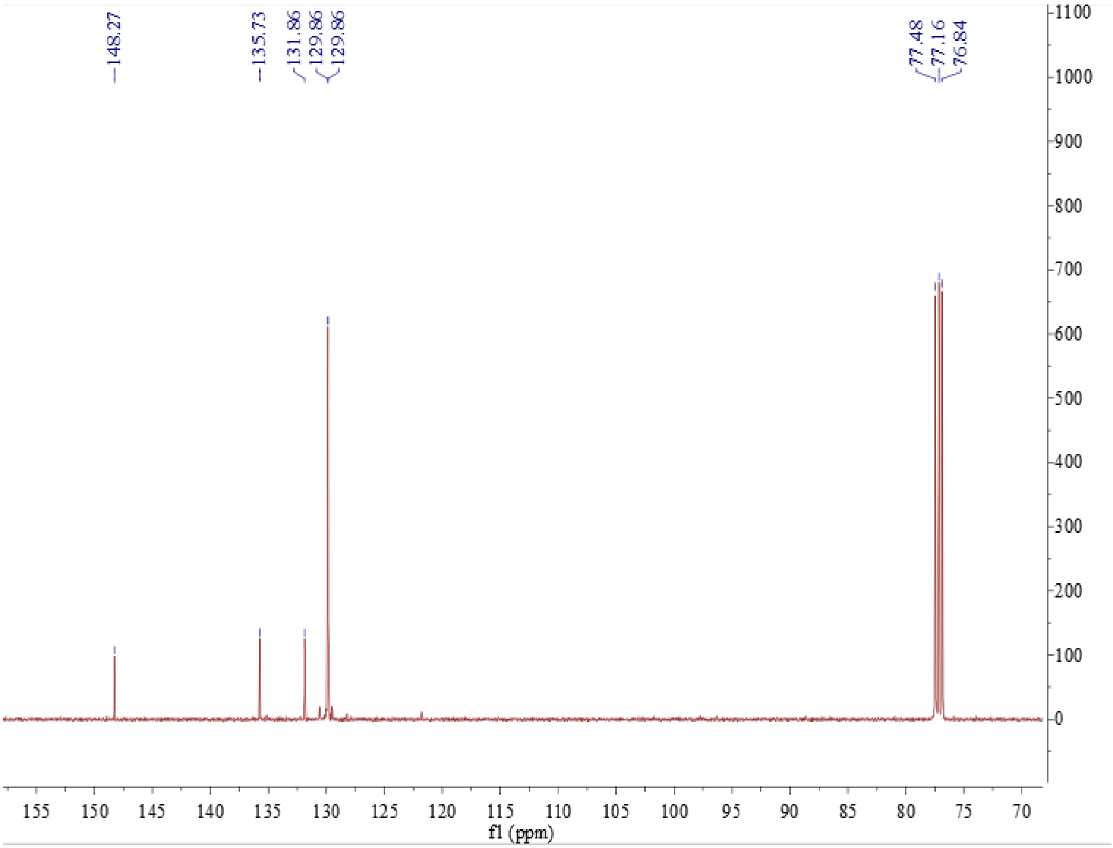

Supplementary Figure 51. ^{13}C NMR spectrum of **11b** in $CDCl_3$ at 25 ºC

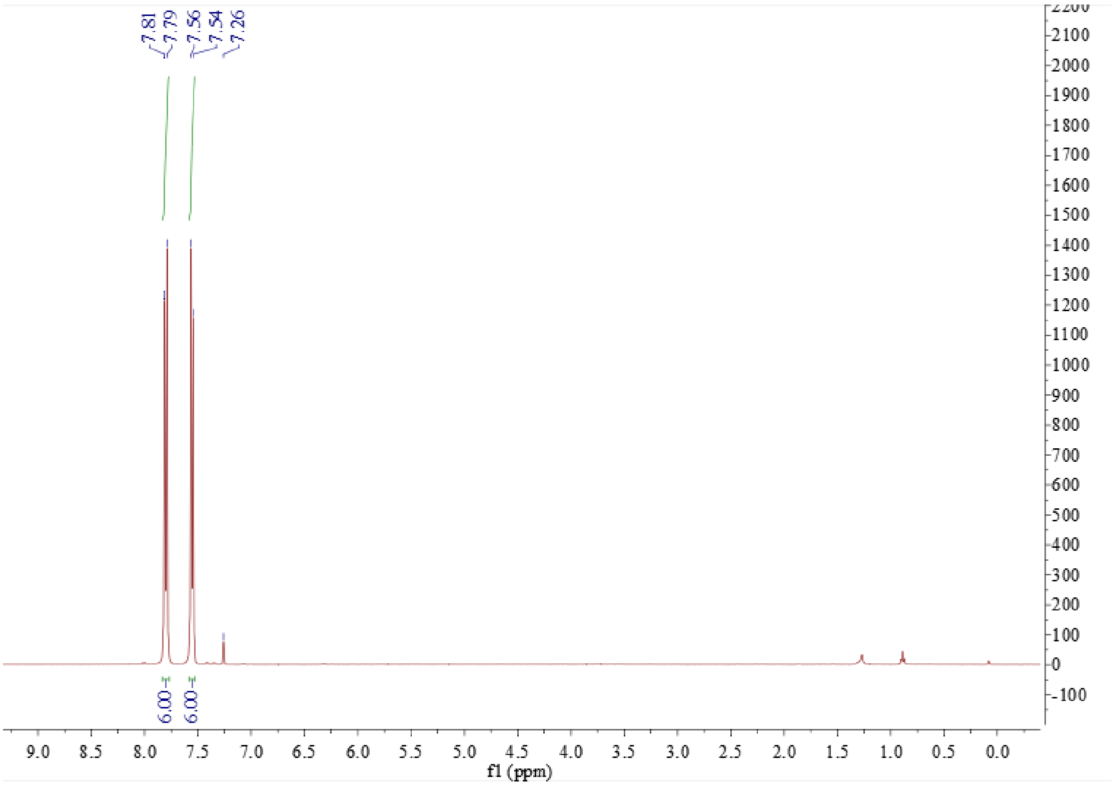

Supplementary Figure 52. ^{1}H NMR spectrum of **11c** in $CDCl_3$ at 25 ºC

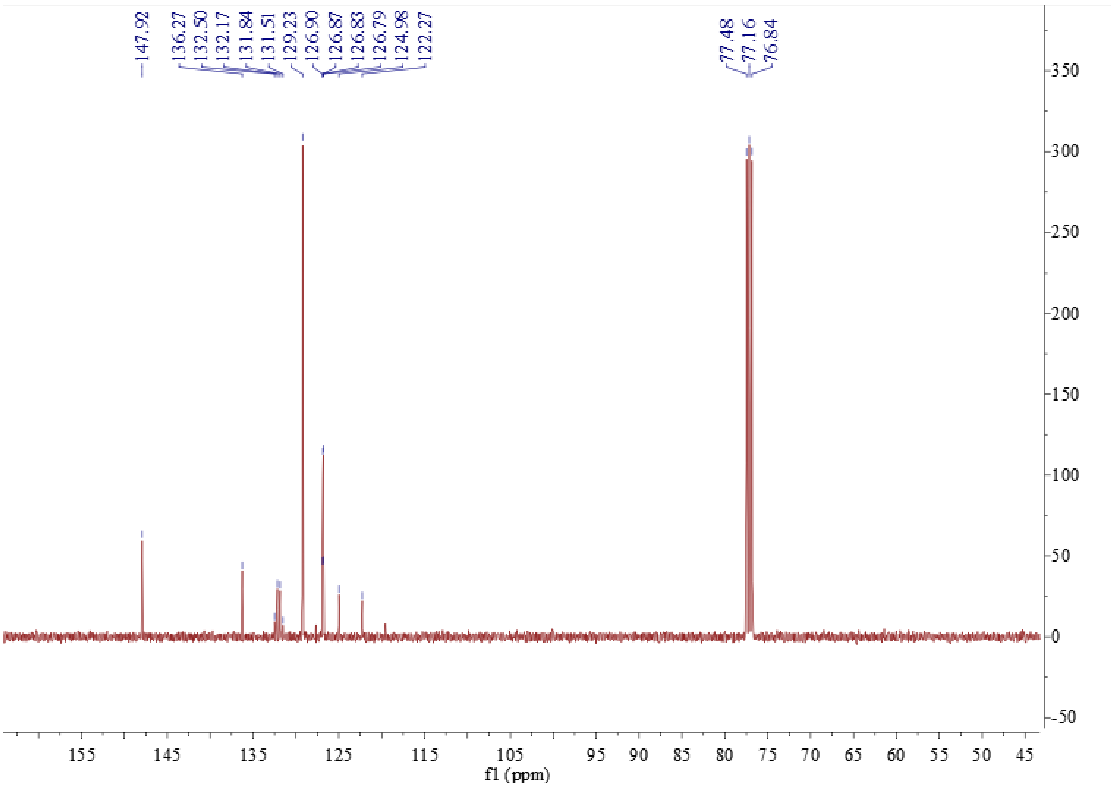

Supplementary Figure 53. ^{13}C NMR spectrum of **11c** in $CDCl_3$ at 25 ºC

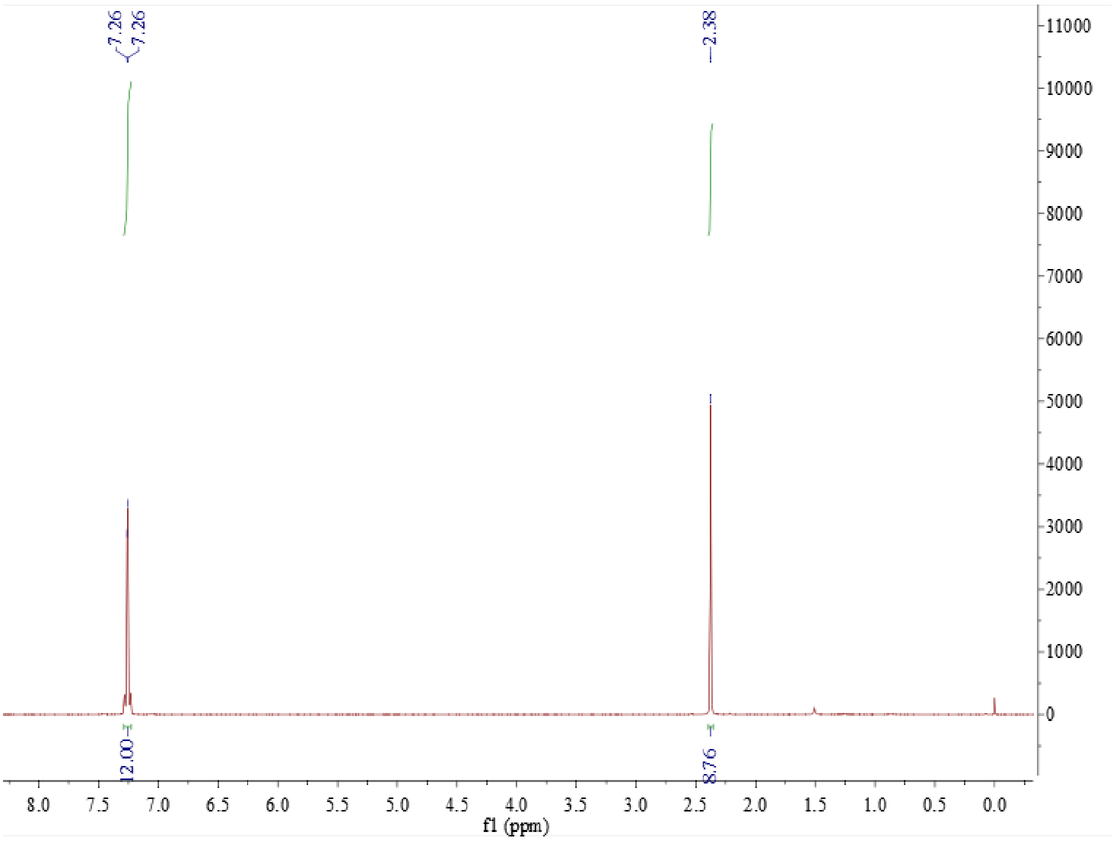

Supplementary Figure 54. ^{1}H NMR spectrum of **11d** in $CDCl_3$ at 25 ºC

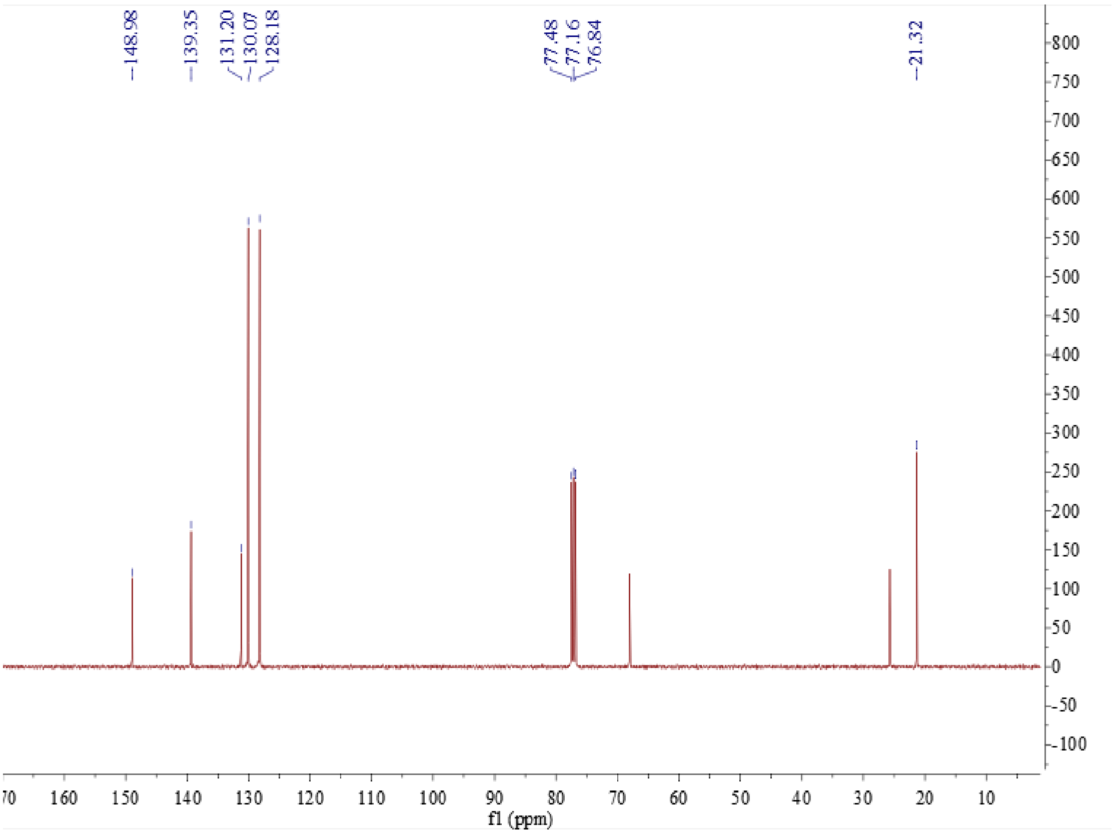

Supplementary Figure 55. ^{13}C NMR spectrum of **11d** in $CDCl_3$ at 25 ºC

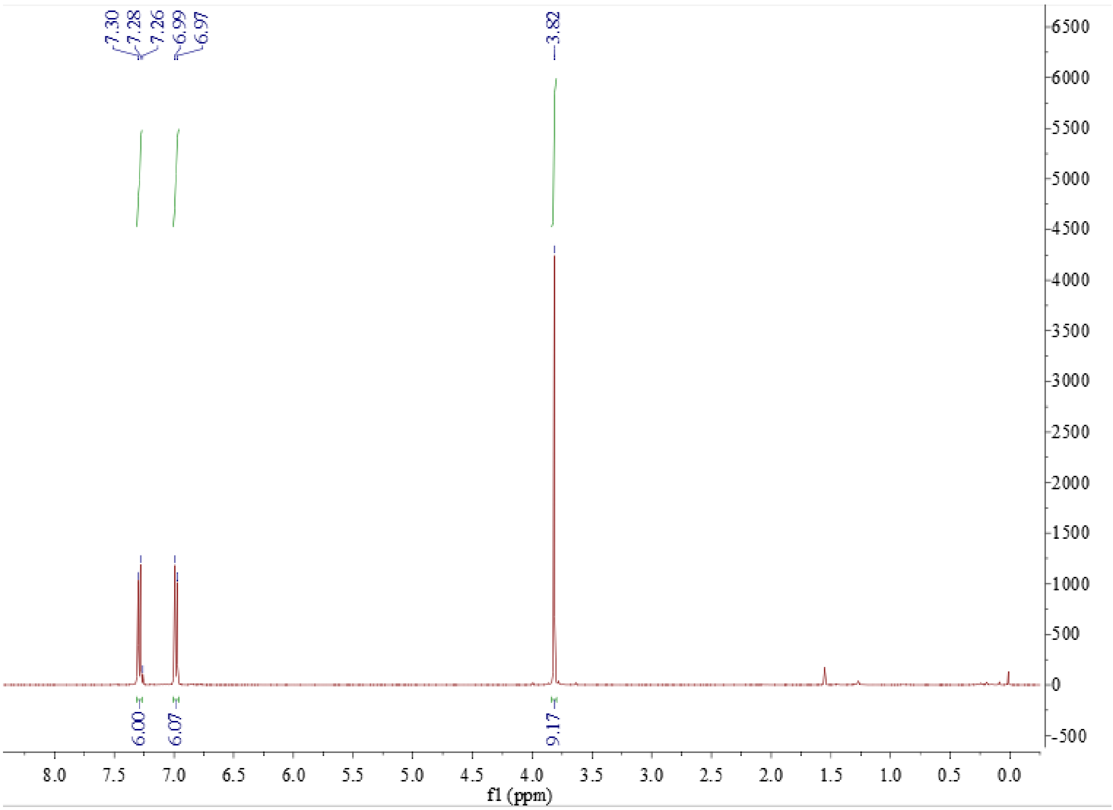

Supplementary Figure 56. ^{1}H NMR spectrum of **11e** in $CDCl_3$ at 25 ºC

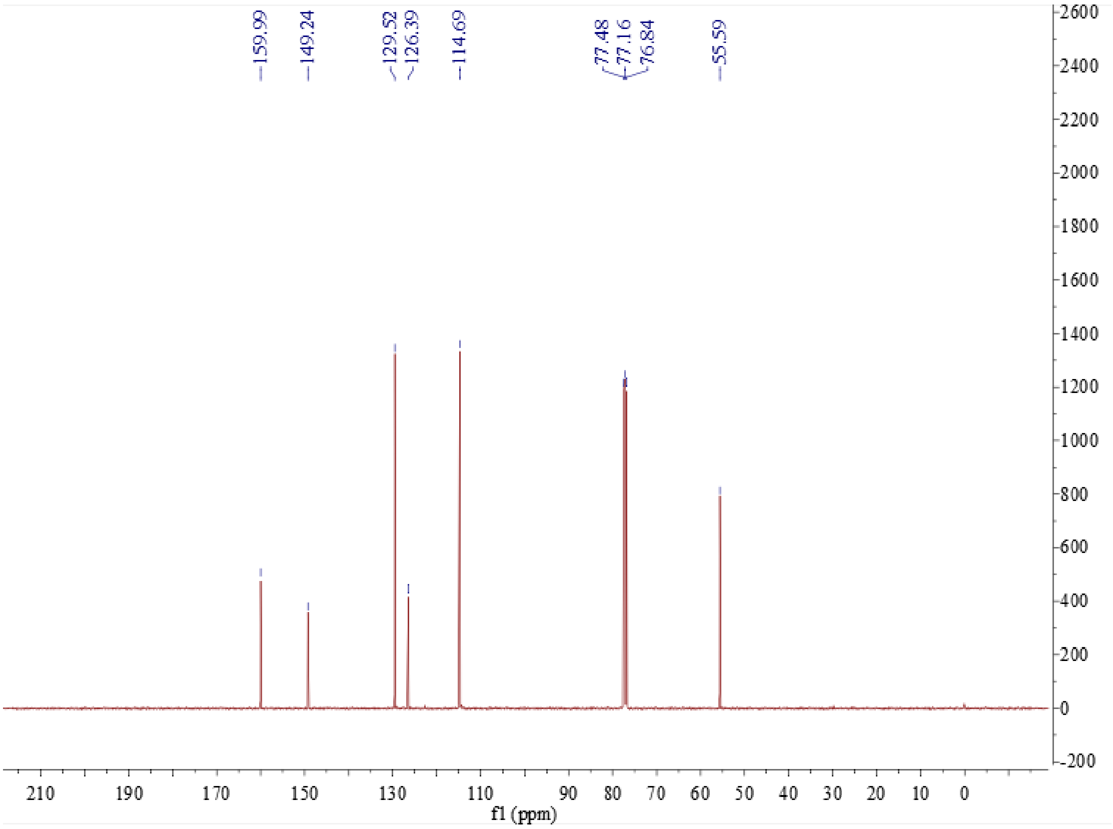

Supplementary Figure 57. ^{13}C NMR spectrum of **11e** in $CDCl_3$ at 25 ºC

Theoretical calculations

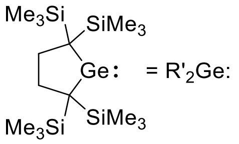

1

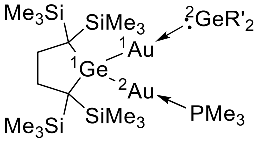

3a

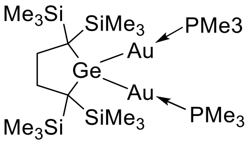

4

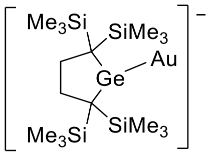

A

Supplementary Table 2. Comparison of Structural Parameters between X-ray structure of **3** and Those Optimized at the B3PW91-GD3 Level

Parameter	X-ray		B3PW91 (**3a**)
	**3a**	**3b**	
Bond distance/Å		—	
^{1}Au-^{1}Ge	2.4475(4)	2.4371(7)	2.44752
^{1}Au -^{2}Ge	2.4146(4)	2.4089(8)	2.41041
^{2}Au -^{1}Ge	2.4460(5)	2.4307(8)	2.44057
^{2}Au -P	2.3298(11)	2.311(2)	2.37245
Bond Angle/deg		—	
^{1}Au -^{1}Ge - ^{2}Au	106.909(16)	107.61(3)	100.02468
^{1}Ge - ^{1}Au-^{2}Ge	168.042(15)	171.73(3)	169.67871
^{1}Ge - ^{2}Au -P	175.25(3)	174.02(7)	174.01159
Dihedral Angle/deg		—	
between two averaged 5-membered ring planes	78.915	84.588	82.522

Supplementary Table 3. Comparison of Frontier MO Energy Levels among **1**, **3a**, and **4**

Compound	HOMO/eV	LUMO/eV
**1**	–5.54	–1.86
**3a**	–4.47	–2.32
**4**	–4.51	–0.56

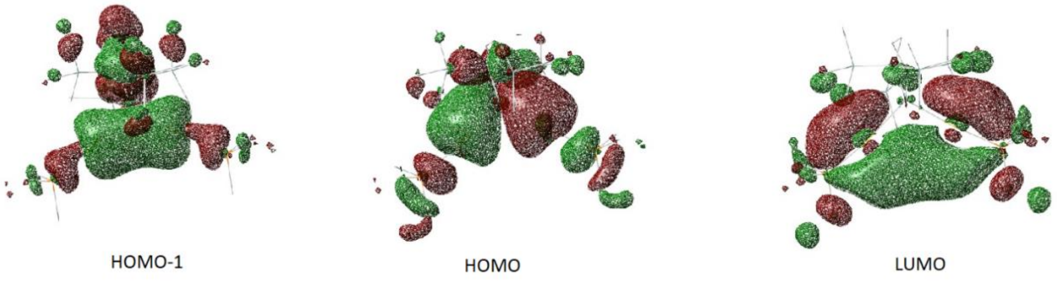

Supplementary Figure 58. Frontier MOs of R'$_2$Ge(AuPMe$_3$)$_2$ (**4**)

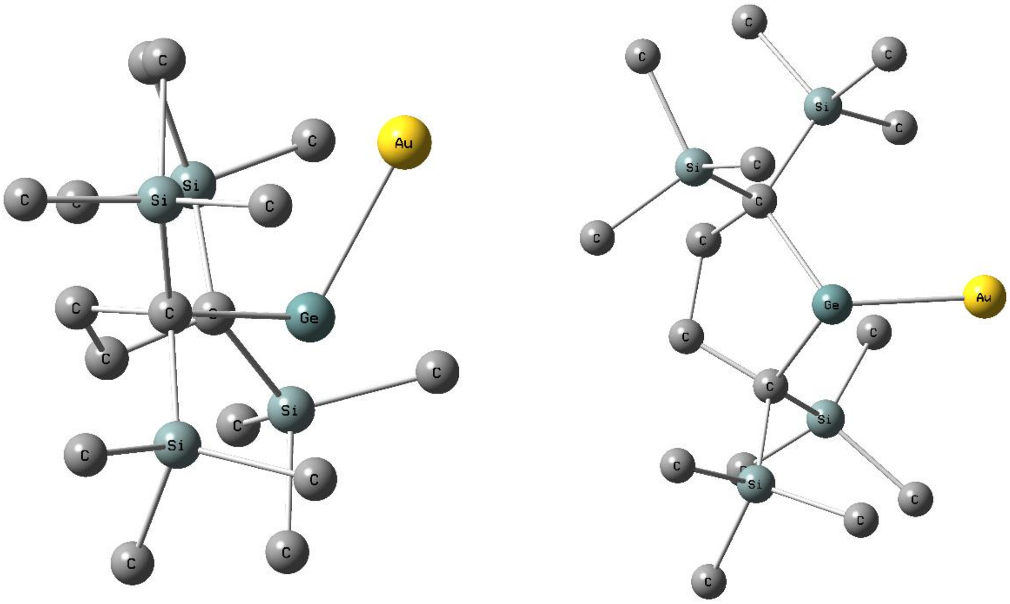

Supplementary Figure 59. Optimized structure of **A**. Hydrogen atoms are omitted for clarity. View from the sideways (left) and top (right).

Supplementary Table 4: Optimized structure of **1** (atom, x-, y-, z- positions in Å).

atom	x	y	z
C	0.678853	0.356288	1.668028
C	-0.67884	-0.35626	1.668051
C	-1.41466	-0.06243	0.335091
Ge	-0.000017	-0.000008	-1.05157
C	1.41464	0.062429	0.335041
Si	2.437448	1.554169	-0.26633
C	3.543473	1.124503	-1.73628
C	3.44418	2.280097	1.156173
C	1.301714	2.94699	-0.88221
Si	2.469017	-1.51874	0.3901
C	4.217854	-1.21275	1.052752
C	2.565248	-2.33825	-1.30998
C	1.758185	-2.77738	1.61247
Si	-2.43742	-1.55417	-0.26633
C	-3.44444	-2.27998	1.156034
C	-1.30167	-2.94713	-0.88194
C	-3.5432	-1.12448	-1.73644
Si	-2.46902	1.518733	0.390128
C	-4.21791	1.212799	1.052678
C	-2.56525	2.338279	-1.30995
C	-1.75814	2.777324	1.612507
H	1.268912	0.075396	2.552557
H	0.494466	1.434004	1.760383
H	-1.26888	-0.07533	2.552582
H	-0.49447	-1.43397	1.760425
H	2.932444	0.813314	-2.59137
H	4.103782	2.017793	-2.03646
H	4.264525	0.329023	-1.53196
H	2.782033	2.606172	1.96604
H	4.004244	3.156462	0.809597
H	4.155124	1.561866	1.572374
H	0.868192	2.72169	-1.86366
H	0.483496	3.153502	-0.18698
H	1.883424	3.870853	-0.98278
H	4.173465	-0.81107	2.071432
H	4.7484	-2.17145	1.09942
H	4.817658	-0.52927	0.446213
H	1.65201	-2.90398	-1.52504
H	3.404893	-3.04185	-1.34521
H	2.701589	-1.60741	-2.11229
H	1.639755	-2.34736	2.612836
H	0.792918	-3.18565	1.304268
H	2.459901	-3.61576	1.696765
H	-4.15604	-1.56198	1.571509
H	-4.0038	-3.15686	0.809593
H	-2.78253	-2.60526	1.966412
H	-0.86882	-2.72237	-1.86381
H	-1.88325	-3.87118	-0.98154
H	-0.48298	-3.15304	-0.1871
H	-4.26443	-0.32917	-1.53212
H	-2.93205	-0.81303	-2.59135
H	-4.10329	-2.01781	-2.03691
H	-4.17363	0.810887	2.07127
H	-4.74836	2.171535	1.099501
H	-4.81773	0.529509	0.44594
H	-1.65207	2.904116	-1.52496
H	-3.40498	3.041782	-1.34519
H	-2.70151	1.607428	-2.11227
H	-1.63954	2.34725	2.612831
H	-0.79295	3.185669	1.304166
H	-2.45992	3.615632	1.696941

Supplementary Table 5: Optimized structure of **3a** (atom, x-, y-, z- positions in Å).

Zero-point correction=	1.147218 (Hartree/Particle)
Thermal correction to Energy=	1.234556
Thermal correction to Enthalpy=	1.235500
Thermal correction to Gibbs Free Energy=	1.020752

Sum of electronic and zero-point Energies= -8466.517605
Sum of electronic and thermal Energies= -8466.430268
Sum of electronic and thermal Enthalpies= -8466.429324
Sum of electronic and thermal Free Energies= -8466.644072

atom	x	y	z
Au	0.580618	-0.46377	0.02986
Ge	-1.90056	-0.73942	0.120997
Ge	2.991133	0.181	-0.05192
Au	-2.68548	1.57178	-0.29234
P	-3.40642	3.832298	-0.73364
C	-2.59401	-1.68302	1.833341
C	-2.81284	-2.11181	-1.14047
C	3.788338	1.987544	0.220983
C	4.752743	-0.69475	-0.37768
C	-5.22848	4.058489	-0.69453
C	-2.9401	4.513587	-2.37471
C	-2.80314	5.108032	0.441826
Si	3.692159	2.62523	2.041487
C	3.852751	4.512398	2.145179
C	5.149185	2.009435	3.085776
C	2.099831	2.088508	2.896442
Si	2.800448	3.154066	-0.96209
C	1.13992	3.708474	-0.25391
C	2.377252	2.284075	-2.59303
C	3.84673	4.661998	-1.42127
Si	-1.58192	-2.70487	-2.48695
C	-0.20668	-3.81326	-1.80442
C	-0.70944	-1.27992	-3.38376
C	-2.37466	-3.78631	-3.84002
Si	-3.3721	-0.4097	3.040511
C	-2.2612	1.089442	3.380237
C	-5.02279	0.300624	2.437726
C	-3.82712	-1.14756	4.737271
Si	-1.271	-2.71133	2.77839
C	-0.02115	-3.69877	1.753917
C	-0.18434	-1.65505	3.92066
C	-2.1006	-4.05756	3.839659
Si	4.80428	-2.2064	0.832063
C	3.911454	-3.72977	0.168764
C	6.594801	-2.63963	1.260478

C	3.948227	-1.78548	2.470603
Si	-4.41568	-1.47843	-1.99941
C	-5.63786	-0.44125	-0.98672
C	-5.49716	-2.94601	-2.5524
C	-4.07294	-0.39917	-3.5241
Si	5.037677	-1.27345	-2.19815
C	5.683697	0.12929	-3.29772
C	6.406651	-2.57984	-2.31457
C	3.462928	-1.94623	-2.98631
C	-3.65391	-2.64258	1.218138
C	-3.17882	-3.23095	-0.12368
C	5.259917	1.811368	-0.25699
C	5.781228	0.39742	0.04373
H	0.595207	-3.04381	1.11979
H	-5.51549	5.102702	-0.89627
H	-5.60965	3.760577	0.29331
H	-5.69157	3.401994	-1.44569
H	-1.84437	4.518781	-2.46825
H	-3.3225	5.536811	-2.51733
H	-3.34363	3.862128	-3.16386
H	-1.70361	5.123277	0.426252
H	-3.18657	6.110016	0.191664
H	-3.12328	4.840386	1.459595
H	4.810246	4.857404	1.723364
H	3.042904	5.066758	1.649969
H	3.843815	4.798122	3.210401
H	5.159394	0.920913	3.237112
H	6.122387	2.3055	2.663769
H	5.07181	2.475336	4.08242
H	2.0704	0.997119	3.043014
H	1.194588	2.365992	2.337674
H	2.03747	2.55441	3.893727
H	0.474157	2.845364	-0.08796
H	1.216102	4.274473	0.68533
H	0.657546	4.362223	-0.99981
H	3.260426	1.901552	-3.1249

H	1.668014	1.451412	-2.45249
H	1.887315	3.00922	-3.26444
H	4.086271	5.298711	-0.55801
H	4.794622	4.366869	-1.89817
H	3.29116	5.276498	-2.14926
H	-0.58932	-4.73866	-1.34692
H	0.413042	-3.29077	-1.06242
H	0.442829	-4.10603	-2.64613
H	-0.15476	-0.65496	-2.66451
H	-1.39476	-0.62707	-3.94082
H	0.01621	-1.69882	-4.10075
H	-2.84754	-4.68706	-3.41644
H	-3.12679	-3.26344	-4.44905
H	-1.57681	-4.12564	-4.52223
H	-2.02087	1.612897	2.440032
H	-2.80215	1.794351	4.034148
H	-1.31531	0.831578	3.875056
H	-5.79908	-0.46891	2.306259
H	-4.91927	0.8506	1.491745
H	-5.38564	1.005576	3.204806
H	-4.5445	-1.97815	4.637102
H	-2.96594	-1.51296	5.315686
H	-4.31771	-0.36256	5.337478
H	-0.48764	-4.45783	1.110342
H	0.647899	-4.22447	2.456281
H	0.317432	-0.8549	3.353736
H	0.597251	-2.29972	4.355354
H	-0.73177	-1.19124	4.753847
H	-2.90767	-3.69023	4.487993
H	-2.5143	-4.85974	3.207134
H	-1.33553	-4.52038	4.485758
H	2.848257	-3.52038	-0.02405
H	4.350571	-4.14455	-0.74957

H	3.951587	-4.51736	0.939301
H	7.11991	-1.79129	1.726736
H	7.179748	-2.96228	0.387621
H	6.595521	-3.46702	1.989592
H	4.377457	-0.90603	2.972654
H	2.864419	-1.62212	2.350143
H	4.064355	-2.63829	3.160284
H	-5.19653	0.509363	-0.65131
H	-6.49463	-0.20979	-1.64281
H	-6.03058	-0.96599	-0.10434
H	-4.96365	-3.71209	-3.13066
H	-5.96146	-3.44121	-1.68402
H	-6.31922	-2.56301	-3.18053
H	-3.44486	0.467111	-3.25991
H	-5.03514	-0.01467	-3.90162
H	-3.5857	-0.93727	-4.35005
H	6.622993	0.561369	-2.91842
H	4.965066	0.947735	-3.44396
H	5.899977	-0.29477	-4.2926
H	7.364061	-2.19286	-1.93063
H	6.183571	-3.51915	-1.78925
H	6.55741	-2.82549	-3.37913
H	3.022263	-2.77757	-2.41792
H	2.688753	-1.16853	-3.08299
H	3.687194	-2.31497	-4.00092
H	-3.91245	-3.46636	1.907632
H	-4.5996	-2.10364	1.039673
H	-3.9548	-3.91069	-0.51963
H	-2.30223	-3.87125	0.072898
H	5.93368	2.560658	0.196506
H	5.330559	1.983665	-1.34443
H	6.760543	0.249409	-0.44644
H	5.989658	0.327262	1.124671

Supplementary Table 6: Optimized structure of **4** (atom, x-, y-, z- positions in Å).

atom	x	y	z
C	0.194513	-3.18867	-0.74104
C	-0.19463	-3.1854	0.754516
C	0.30805	-1.89478	1.458024
Ge	-0.000015	-0.49569	0.000867
C	-0.30803	-1.90107	-1.45019

Au	1.906313	1.006322	-0.21936
Au	-1.90643	1.007145	0.214983
Si	0.708191	-1.37791	-2.96494
C	0.433205	0.455171	-3.39976
C	0.328524	-2.37544	-4.54074
C	2.569436	-1.63861	-2.74489

Si	-2.13057	-2.07752	-1.96571
C	-2.39052	-3.73771	-2.85611
C	-2.70237	-0.66725	-3.09567
C	-3.46268	-2.13747	-0.61887
Si	-0.7081	-1.36494	2.970474
C	-0.32847	-2.35554	4.550641
C	-2.56934	-1.62668	2.751557
C	-0.43294	0.469995	3.397248
Si	2.130636	-2.069	1.974173
C	2.390816	-3.72518	2.871931
C	2.702578	-0.65387	3.098012
C	3.462497	-2.13482	0.627365
P	-3.91084	2.269273	0.327097
C	-3.90564	4.103389	0.236612
C	-5.07757	1.784037	-1.0028
C	-4.86315	1.924779	1.857206
P	3.910833	2.267877	-0.33598
C	5.077259	1.78716	0.995829
C	3.905887	4.102302	-0.25193
C	4.863392	1.917871	-1.86468
H	-0.19053	-4.10016	-1.22062
H	1.287761	-3.25558	-0.80813
H	0.190299	-4.09484	1.238063
H	-1.28788	-3.2519	0.821905
H	-0.29166	0.934988	-2.73564
H	1.376659	1.003506	-3.2969
H	0.081393	0.569056	-4.43185
H	0.523867	-3.44402	-4.39445
H	0.995622	-2.02456	-5.3384
H	-0.70028	-2.26439	-4.8966
H	2.831694	-2.68641	-2.56587
H	3.06337	-1.3252	-3.67322
H	2.968418	-1.03787	-1.92496
H	-2.43012	-4.55313	-2.12411
H	-3.35585	-3.72253	-3.37698
H	-1.61578	-3.98095	-3.58612
H	-3.76425	-0.81545	-3.32714
H	-2.157	-0.60035	-4.04083
H	-2.59944	0.292772	-2.57734

H	-3.284	-2.90476	0.138542
H	-3.57053	-1.17706	-0.10737
H	-4.41311	-2.37305	-1.11592
H	-0.52337	-3.42481	4.408938
H	0.700224	-2.24255	4.906224
H	-0.99585	-2.00146	5.346647
H	-2.96828	-1.03026	1.928456
H	-3.0634	-1.30847	3.678184
H	-2.83149	-2.67543	2.577995
H	-1.37679	1.017569	3.294008
H	-0.07915	0.588264	4.428166
H	0.290459	0.947295	2.729717
H	2.428617	-4.54411	2.143778
H	3.357035	-3.70819	3.391093
H	1.617142	-3.96435	3.604406
H	3.764561	-0.80096	3.329735
H	2.157541	-0.58326	4.043102
H	2.59932	0.304044	2.575883
H	3.284196	-2.90614	-0.12606
H	3.569451	-1.17699	0.110877
H	4.413249	-2.36712	1.12533
H	-3.3105	4.503665	1.061948
H	-3.44175	4.416166	-0.70283
H	-4.92212	4.50878	0.291945
H	-4.64486	2.036766	-1.9744
H	-6.04587	2.283427	-0.89
H	-5.21585	0.699916	-0.97107
H	-4.99972	0.844026	1.951824
H	-4.29262	2.269592	2.723837
H	-5.84061	2.41891	1.842246
H	4.644424	2.043424	1.966449
H	6.045672	2.285961	0.881413
H	5.215319	0.702903	0.967996
H	3.441756	4.418433	0.686272
H	4.922449	4.507326	-0.30836
H	3.311066	4.499795	-1.07884
H	5.840866	2.412021	-1.8513
H	4.999963	0.836779	-1.95538
H	4.293037	2.259587	-2.73265

Supplementary Table 7: Optimized structure of **A** (atom, x-, y-, z- positions in Å).

atom	x	y	z
C	-0.93626	1.335103	1.760061
C	0.392552	2.02039	1.381237
C	1.302524	1.076439	0.553369
Ge	-0.07157	0.262964	-0.82675
C	-1.63316	0.695208	0.528786
Au	0.425659	-2.17797	-0.88573
Si	-2.70661	-0.76828	1.056536
C	-3.62434	-1.53843	-0.41045
C	-3.98507	-0.25441	2.386253
C	-1.83181	-2.19589	1.931201
Si	-2.68579	1.936672	-0.45543
C	-4.44581	2.212113	0.230951
C	-1.98442	3.700913	-0.44673
C	-2.93572	1.466948	-2.27645
Si	2.453336	1.973419	-0.65147
C	3.985715	2.783719	0.152069
C	3.097338	0.823693	-2.02227
C	1.616464	3.438392	-1.52463
Si	2.329825	-0.04401	1.676208
C	3.14164	0.99705	3.060999
C	1.436582	-1.38304	2.663924
C	3.719271	-0.96232	0.772172
H	-1.58224	2.065524	2.278442
H	-0.71666	0.557494	2.506017
H	0.885926	2.388345	2.295301
H	0.153304	2.920081	0.802112
H	-4.17113	-2.42861	-0.07618
H	-2.89491	-1.85143	-1.16715
H	-4.34152	-0.85568	-0.87785
H	-4.2941	-1.14365	2.949896
H	-3.53263	0.446659	3.098698
H	-4.88429	0.215714	1.981452
H	-1.04967	-2.64478	1.311803
H	-1.39473	-1.89242	2.886846
H	-2.5935	-2.9596	2.137807
H	-4.89073	3.057019	-0.31098
H	-5.09682	1.344123	0.086247
H	-4.43896	2.460167	1.297864
H	-2.69623	4.354939	-0.96701
H	-1.02423	3.773222	-0.96094
H	-1.86067	4.083972	0.572827
H	-3.06135	0.38908	-2.41275
H	-3.82841	1.974466	-2.66653
H	-2.06962	1.765828	-2.87448
H	4.536426	3.33897	-0.61883
H	3.695062	3.495462	0.934064
H	4.672298	2.055051	0.595356
H	2.58625	1.05384	-2.96332
H	2.900547	-0.22967	-1.80343
H	4.177071	0.95228	-2.17075
H	1.286423	4.215491	-0.82636
H	2.344314	3.891774	-2.20999
H	0.756816	3.103581	-2.11412
H	3.876427	0.377489	3.591445
H	3.650284	1.894602	2.699922
H	2.388829	1.309223	3.795127
H	2.178763	-1.88699	3.297555
H	0.981465	-2.12555	2.004978
H	0.662117	-0.97089	3.318177
H	3.285602	-1.65087	0.038047
H	4.427808	-0.30708	0.25668
H	4.276062	-1.55692	1.507487

Supplementary Table 8: Optimized structure of **MeOTf** (atom, x-, y-, z- positions in Å).

Zero-point correction=	0.067515 (Hartree/Particle)
Thermal correction to Energy=	0.076902
Thermal correction to Enthalpy=	0.077846
Thermal correction to Gibbs Free Energy=	0.031682
Sum of electronic and zero-point Energies=	-1000.399501

Sum of electronic and thermal Energies= -1000.390114
Sum of electronic and thermal Enthalpies= -1000.389170
Sum of electronic and thermal Free Energies= -1000.435334

atom	x	y	z
C	2.251547	1.204049	-0.04399
O	1.425607	0.26684	-0.7521
S	0.417642	-0.69613	0.055876
O	0.809718	-0.7833	1.449388
O	0.176625	-1.84423	-0.78225
C	-1.12492	0.356033	-0.00417
F	-1.51525	0.519515	-1.25407
F	-2.07711	-0.24445	0.686918
F	-0.8676	1.542267	0.536712
H	1.731865	2.167957	0.050101
H	2.512574	0.815208	0.949332
H	3.157467	1.323901	-0.65084

Supplementary Table 9: Optimized structure of **Int1** (atom, x-, y-, z- positions in Å).

Zero-point correction= 1.216578 (Hartree/Particle)
Thermal correction to Energy= 1.314095
Thermal correction to Enthalpy= 1.315039
Thermal correction to Gibbs Free Energy= 1.079982
Sum of electronic and zero-point Energies= -9466.913291
Sum of electronic and thermal Energies= -9466.815773
Sum of electronic and thermal Enthalpies= -9466.814829
Sum of electronic and thermal Free Energies= -9467.049886

atom	x	y	z
Au	0.249601	-1.21585	0.619267
Ge	-2.19204	-1.05161	0.466949
Ge	2.73038	-0.77813	0.225544
Au	-0.96382	0.62343	-1.15416
P	-1.11597	2.20146	-2.99648
C	-3.44424	-0.41125	1.905223
C	-3.53069	-2.26965	-0.43876
C	4.025292	-1.6845	-1.00339
C	4.116376	0.378856	1.077475
C	-2.37591	3.513508	-2.85528
C	-1.54479	1.352866	-4.56917
C	0.421946	3.086843	-3.42434
Si	3.702721	-1.30517	-2.87024
C	4.661335	-2.47599	-4.01114
C	4.318037	0.414657	-3.35809
C	1.869024	-1.40578	-3.31179
Si	4.007267	-3.59684	-0.69294
C	2.639977	-4.51772	-1.60842
C	3.777967	-3.99373	1.142539
C	5.674214	-4.35062	-1.1779
Si	-2.82057	-4.04452	-0.74588
C	-2.52526	-5.01245	0.848758
C	-1.15979	-4.00251	-1.6416
C	-4.00093	-5.15412	-1.73294
Si	-3.2936	1.530839	2.069668
C	-1.50917	2.043724	2.324483
C	-3.9254	2.385579	0.516033
C	-4.32212	2.263385	3.480326
Si	-3.25008	-1.20228	3.674726
C	-3.16394	-3.09422	3.751918
C	-1.7458	-0.56482	4.621445
C	-4.80002	-0.86239	4.715735
Si	3.378961	2.177707	1.029255
C	2.072221	2.424844	2.351958
C	4.726032	3.497151	1.159584
C	2.606587	2.441344	-0.672
Si	-4.25578	-1.6236	-2.12322

C	-4.57796	0.23334	-2.23651
C	-5.96995	-2.38048	-2.42026
C	-3.15742	-2.02427	-3.61116
Si	4.659621	-0.08892	2.872565
C	6.111686	-1.30957	2.89126
C	5.340107	1.393167	3.834099
C	3.247184	-0.83919	3.873616
C	-4.8031	-0.88673	1.308617
C	-4.67346	-2.25908	0.624636
C	5.40851	-1.12796	-0.53951
C	5.293547	0.276802	0.064678
H	-2.34254	-3.55256	3.188931
H	-2.33558	4.152734	-3.7514
H	-2.18252	4.120145	-1.95779
H	-3.37689	3.067509	-2.77674
H	-0.78648	0.591512	-4.80264
H	-1.59004	2.080294	-5.39506
H	-2.51909	0.854256	-4.47882
H	1.240032	2.373619	-3.59398
H	0.261246	3.686337	-4.33392
H	0.667262	3.741506	-2.57853
H	5.746843	-2.41508	-3.83436
H	4.361302	-3.53081	-3.93524
H	4.483283	-2.15675	-5.05172
H	3.780538	1.225063	-2.8477
H	5.395026	0.543392	-3.16742
H	4.163642	0.542241	-4.4427
H	1.268099	-0.67367	-2.74675
H	1.438948	-2.39906	-3.11987
H	1.744083	-1.18874	-4.38577
H	1.641765	-4.15181	-1.3298
H	2.727648	-4.46524	-2.70315
H	2.691744	-5.58201	-1.32467
H	4.571816	-3.5651	1.770188
H	2.80854	-3.65462	1.536679
H	3.815551	-5.08773	1.27618
H	5.911252	-4.23486	-2.24453
H	6.506599	-3.92525	-0.59652
H	5.641227	-5.43105	-0.95852
H	-3.42942	-5.13412	1.46283
H	-1.73664	-4.57291	1.473986
H	-2.18904	-6.0223	0.559599

H	-1.21824	-3.64023	-2.67579
H	-0.74471	-5.023	-1.66595
H	-0.4453	-3.36251	-1.09991
H	-4.96565	-5.28304	-1.21688
H	-4.20397	-4.81142	-2.75738
H	-3.53585	-6.15152	-1.80643
H	-1.0439	1.593218	3.210009
H	-0.88967	1.799631	1.447604
H	-1.45186	3.138358	2.433601
H	-5.01772	2.319974	0.392225
H	-3.44736	1.995064	-0.39281
H	-3.64317	3.449941	0.580773
H	-5.38541	1.981464	3.431569
H	-3.93999	2.023731	4.483368
H	-4.26486	3.358653	3.363252
H	-4.10366	-3.56608	3.426976
H	-3.02154	-3.36148	4.812784
H	-0.81174	-0.6713	4.049337
H	-1.64219	-1.15915	5.544094
H	-1.84633	0.48938	4.917517
H	-5.04517	0.197497	4.850574
H	-5.68733	-1.36984	4.304925
H	-4.62067	-1.29442	5.714881
H	1.331621	1.611711	2.333782
H	2.489929	2.481026	3.368104
H	1.520205	3.359351	2.154903
H	5.542973	3.327336	0.44048
H	5.162284	3.595246	2.162478
H	4.257925	4.462798	0.903776
H	3.364538	2.691198	-1.42969
H	2.013232	1.582502	-1.03451
H	1.890962	3.275196	-0.59487
H	-3.65798	0.826236	-2.13786
H	-5.00979	0.439062	-3.23069
H	-5.29439	0.59703	-1.48702
H	-6.02358	-3.46903	-2.28934
H	-6.72693	-1.92002	-1.76575
H	-6.26481	-2.15411	-3.45886
H	-2.14274	-1.61364	-3.49355
H	-3.60811	-1.55728	-4.50246
H	-3.06926	-3.09997	-3.81854
H	6.989406	-0.91524	2.355295

H	5.879361	-2.30107	2.479051	H	6.254473	0.558058	0.530863
H	6.414493	-1.45678	3.941458	H	5.148848	1.000776	-0.75596
H	6.215919	1.83721	3.33614	C	0.340644	-2.79046	2.061277
H	4.605125	2.190566	4.012041	H	1.149809	-2.55613	2.763591
H	5.673928	1.02558	4.819003	H	-0.58767	-2.90769	2.627449
H	2.336958	-0.22217	3.841411	H	0.562298	-3.72441	1.526571
H	2.990747	-1.8484	3.515945	O	-0.16676	3.903989	-0.49409
H	3.550256	-0.93462	4.929095	S	-0.68783	5.070187	0.275751
H	-5.58545	-0.93841	2.08442	O	-2.10794	5.364812	0.009974
H	-5.17313	-0.15523	0.570394	O	-0.25135	5.134051	1.677968
H	-5.64146	-2.54094	0.178696	C	0.212804	6.492695	-0.52274
H	-4.47484	-3.01671	1.398861	F	-0.04282	6.535363	-1.83715
H	6.139732	-1.11939	-1.36778	F	-0.1507	7.654589	0.008162
H	5.847573	-1.79271	0.222981	F	1.53504	6.358043	-0.37272

Supplementary Table 10: Optimized structure of **Int2** (atom, x-, y-, z- positions in Å).

Zero-point correction=	1.215263 (Hartree/Particle)
Thermal correction to Energy=	1.313198
Thermal correction to Enthalpy=	1.314142
Thermal correction to Gibbs Free Energy=	1.076738
Sum of electronic and zero-point Energies=	-9466.942465
Sum of electronic and thermal Energies=	-9466.844530
Sum of electronic and thermal Enthalpies=	-9466.843586
Sum of electronic and thermal Free Energies=	-9467.080990

atom	x	y	z				
				C	-3.30417	1.151702	-3.43963
Au	-0.81026	0.247077	-0.26815	Si	-3.04037	3.986458	-0.51906
Ge	2.601245	0.83688	0.614389	C	-1.70927	4.065729	-1.85273
Ge	-3.03463	0.94432	0.201305	C	-2.14635	4.069293	1.148628
Au	1.583283	-0.49051	-1.26389	C	-4.14076	5.520404	-0.58301
P	1.017383	-1.76104	-3.306	Si	4.222284	3.58348	-0.27502
C	3.529479	-0.25244	2.067161	C	4.299333	4.508863	1.376685
C	4.349936	1.667687	-0.09842	C	2.624011	4.114507	-1.16024
C	-4.10808	2.372905	-0.63428	C	5.672405	4.370529	-1.22198
C	-4.38813	0.343331	1.479184	Si	2.860271	-2.06363	2.076628
C	-0.74302	-1.88012	-3.76339	C	0.969139	-2.08938	2.179417
C	1.604096	-3.48979	-3.32435	C	3.383947	-3.03297	0.539527
C	1.799516	-1.00896	-4.78985	C	3.555414	-3.11929	3.495667
Si	-4.65497	2.03162	-2.46255	Si	3.424875	0.422215	3.879593
C	-5.12372	3.627859	-3.36601	C	3.539195	2.294031	4.1765
C	-6.21973	0.974946	-2.53839	C	1.830445	-0.07343	4.776281

C	4.905906	-0.23227	4.875527
Si	-4.46934	-1.58719	1.157326
C	-3.08139	-2.49296	2.029879
C	-6.15829	-2.25741	1.669448
C	-4.31889	-1.8921	-0.70295
Si	4.970767	0.966778	-1.78127
C	4.823549	-0.90715	-2.04858
C	6.838882	1.237548	-1.99182
C	4.063365	1.738839	-3.25651
Si	-4.04097	0.7343	3.340087
C	-4.65079	2.465585	3.811978
C	-5.0065	-0.42156	4.482408
C	-2.20408	0.631335	3.748321
C	5.002561	-0.16538	1.570776
C	5.334105	1.236794	1.032473
C	-5.32982	2.44367	0.335389
C	-5.65933	1.068354	0.945407
H	2.646801	2.84972	3.856655
H	-0.85203	-2.3979	-4.72857
H	-1.18139	-0.87532	-3.82769
H	-1.24569	-2.46693	-2.9833
H	2.651747	-3.54552	-2.997
H	1.511462	-3.90894	-4.33838
H	0.963401	-4.07547	-2.64922
H	2.891306	-0.9784	-4.66673
H	1.556396	-1.59529	-5.69007
H	1.43913	0.021253	-4.92504
H	-5.94791	4.155919	-2.8613
H	-4.2924	4.334976	-3.49855
H	-5.48207	3.350099	-4.37139
H	-6.07477	-0.04354	-2.1532
H	-7.06522	1.432595	-2.00161
H	-6.51735	0.885344	-3.59648
H	-3.14765	0.129426	-3.06256
H	-2.33973	1.678763	-3.40527
H	-3.60821	1.068974	-4.49603
H	-1.03032	3.200493	-1.78816
H	-2.11009	4.11087	-2.87566
H	-1.1019	4.972045	-1.6956
H	-2.84333	4.115727	1.997963
H	-1.44952	3.232597	1.317763
H	-1.54199	4.991282	1.177662
H	-4.63761	5.663633	-1.55197
H	-4.9166	5.495406	0.198208
H	-3.51394	6.407668	-0.39301
H	5.2674	4.369308	1.883338
H	3.505065	4.2477	2.087346
H	4.203928	5.585308	1.154777
H	2.832849	4.933908	-1.86693
H	1.884489	4.487533	-0.43558
H	2.153766	3.297704	-1.72663
H	6.635191	4.182402	-0.72093
H	5.767007	4.057655	-2.27156
H	5.512721	5.462167	-1.21802
H	0.629743	-2.8087	2.941548
H	0.551017	-1.10673	2.442679
H	0.521588	-2.4043	1.223031
H	4.474693	-3.15801	0.456427
H	3.011042	-2.58577	-0.39421
H	2.932756	-4.03513	0.609647
H	4.653903	-3.19382	3.45212
H	3.275471	-2.77552	4.501883
H	3.154357	-4.13961	3.373868
H	4.423059	2.760038	3.719177
H	3.63051	2.430376	5.26772
H	0.941202	0.382357	4.314334
H	1.89213	0.306365	5.809865
H	1.659	-1.15749	4.830764
H	5.090352	-1.30871	4.763043
H	5.83117	0.30133	4.603996
H	4.722679	-0.03361	5.944822
H	-2.09697	-2.0802	1.763794
H	-3.17888	-2.5101	3.125016
H	-3.07841	-3.52729	1.650265
H	-6.98619	-1.7155	1.185965
H	-6.3235	-2.24268	2.755635
H	-6.21189	-3.30736	1.335978
H	-5.24699	-1.66599	-1.2481
H	-3.48124	-1.35908	-1.18746
H	-4.08178	-2.96261	-0.82995
H	3.787427	-1.28807	-2.04378
H	5.24702	-1.13637	-3.04142
H	5.387236	-1.49234	-1.30797
H	7.178864	2.266133	-1.81682

H	7.408776	0.574059	-1.32159
H	7.11662	0.964567	-3.0238
H	2.985087	1.514984	-3.21879
H	4.462213	1.305531	-4.18849
H	4.17425	2.830137	-3.32691
H	-5.72335	2.606054	3.60503
H	-4.0973	3.278938	3.321925
H	-4.5128	2.588719	4.899199
H	-6.09106	-0.35691	4.30214
H	-4.70763	-1.47606	4.402136
H	-4.82668	-0.10416	5.52313
H	-1.76985	-0.34169	3.477759
H	-1.62972	1.416631	3.231305
H	-2.0558	0.776338	4.830793
H	5.718651	-0.43273	2.366251
H	5.177337	-0.89798	0.764828
H	6.383639	1.26377	0.690369

H	5.282923	1.947737	1.874303
H	-6.22264	2.846707	-0.17336
H	-5.12176	3.147881	1.159064
H	-6.41378	1.190114	1.741604
H	-6.14929	0.452433	0.172599
C	1.369229	2.180849	1.387678
H	0.676298	1.66419	2.06865
H	1.892105	2.962611	1.951145
H	0.783497	2.652852	0.588766
O	-0.8811	-3.11855	-0.61435
S	-1.41658	-4.4756	-0.91268
O	-2.7685	-4.7463	-0.39081
O	-1.15842	-4.92384	-2.2937
C	-0.34678	-5.60646	0.113802
F	-0.4141	-5.28957	1.408663
F	-0.72824	-6.87165	-0.02631
F	0.936578	-5.51734	-0.26297

Supplementary Table 11: Optimized structure of **Me_3PAuOTf** (atom, x-, y-, z-positions in Å).

Zero-point correction=	0.143633 (Hartree/Particle)
Thermal correction to Energy=	0.159916
Thermal correction to Enthalpy=	0.160861
Thermal correction to Gibbs Free Energy=	0.096747
Sum of electronic and zero-point Energies=	-1557.214192
Sum of electronic and thermal Energies=	-1557.197908
Sum of electronic and thermal Enthalpies=	-1557.196964
Sum of electronic and thermal Free Energies=	-1557.261078

atom	x	y	z
O	1.226078	0.720797	0.799247
S	2.290239	0.747931	-0.32043
O	1.683668	0.567393	-1.64058
O	3.271752	1.794987	-0.10244
C	3.179504	-0.85205	0.028188
F	3.741278	-0.82849	1.229199
F	2.323486	-1.87479	-0.02162
F	4.122771	-1.0463	-0.88525
Au	-0.72518	0.235122	0.293225
P	-2.85043	-0.27521	-0.20409
C	-3.44947	-1.81832	0.569368

C	-3.11643	-0.50823	-1.99571
C	-4.06018	1.000395	0.293497
H	-3.38891	-1.72735	1.66347
H	-2.80718	-2.6546	0.257649
H	-4.49004	-2.02654	0.276147
H	-2.46404	-1.31582	-2.35795
H	-2.84176	0.414444	-2.52698
H	-4.16705	-0.75846	-2.20926
H	-3.80666	1.952473	-0.1947
H	-5.0821	0.704431	0.010315
H	-4.0105	1.147999	1.381955

Supplementary Table 12: Optimized structure of **5** (atom, x-, y-, z- positions in Å).

Zero-point correction=	1.070488 (Hartree/Particle)
Thermal correction to Energy=	1.149142
Thermal correction to Enthalpy=	1.150086
Thermal correction to Gibbs Free Energy=	0.958034
Sum of electronic and zero-point Energies=	-7909.733039
Sum of electronic and thermal Energies=	-7909.654386
Sum of electronic and thermal Enthalpies=	-7909.653442
Sum of electronic and thermal Free Energies=	-7909.845494

atom	x	y	z
Au	-0.000097	0.033756	0.063027
Ge	-2.49059	0.043854	0.070738
Ge	2.4671	0.018848	0.443598
C	-3.98827	0.018427	-1.2372
C	-3.67854	0.108561	1.658886
C	3.673038	1.403504	-0.48449
C	2.605775	0.237297	2.4204
C	3.497703	-1.58997	-0.31628
Si	2.602168	2.765348	-1.31704
C	1.35719	3.559624	-0.12848
C	1.582946	2.115024	-2.77445
C	3.611367	4.179696	-2.09224
Si	-3.59183	1.439881	-2.49269
C	-5.18803	2.094599	-3.26536
C	-2.37739	0.927233	-3.84211
C	-2.78395	2.909804	-1.60781
Si	2.531854	-2.51331	-1.70652
C	1.534433	-1.45945	-2.92573
C	1.257126	-3.74228	-1.02538
C	3.726057	-3.45086	-2.85239
Si	-4.22742	-1.66156	-2.16271
C	-5.22304	-1.44721	-3.76103
C	-5.27271	-2.88365	-1.1602
C	-2.5825	-2.49326	-2.56006
Si	-3.06795	-1.33499	2.798729
C	-4.47354	-1.93121	3.913094
C	-2.53442	-2.83198	1.763339
C	-1.55562	-0.87558	3.827447
Si	-3.6446	1.788704	2.615484
C	-1.92507	2.562802	2.641849

C	-4.83743	3.053547	1.860905
C	-4.2718	1.598285	4.393545
Si	3.993427	-2.8704	1.031548
C	5.440853	-2.31072	2.120088
C	2.576948	-3.30801	2.214076
C	4.639188	-4.51326	0.31872
Si	5.023842	2.268417	0.593758
C	4.379588	3.763917	1.570048
C	5.950253	1.255794	1.902934
C	6.441659	2.872645	-0.52295
C	4.371016	0.488223	-1.53591
C	4.74089	-0.8782	-0.93296
C	-5.22689	0.370333	-0.35861
C	-5.08751	-0.19036	1.065814
H	1.798797	-0.32234	2.913206
H	2.460536	1.302994	2.6557
H	3.564393	-0.08537	2.845297
H	0.684121	2.797308	0.297001
H	0.73854	4.281481	-0.68746
H	1.826045	4.098786	0.705655
H	0.993858	2.95478	-3.17932
H	0.881046	1.325078	-2.47185
H	2.206182	1.726914	-3.59471
H	4.196703	4.766943	-1.36984
H	2.909738	4.871845	-2.58779
H	4.302269	3.807181	-2.86568
H	-5.71808	1.339979	-3.86327
H	-4.94067	2.937862	-3.93156
H	-5.88474	2.473766	-2.50138
H	-2.17524	1.804783	-4.47823
H	-2.74506	0.124138	-4.4963

H	-1.41392	0.607912	-3.41628
H	-3.39588	3.311349	-0.78679
H	-2.64542	3.72733	-2.3351
H	-1.78438	2.668576	-1.20937
H	2.149638	-0.74162	-3.48626
H	0.723341	-0.90451	-2.42974
H	1.076389	-2.14579	-3.65806
H	0.71638	-4.19095	-1.87483
H	0.515081	-3.22412	-0.39697
H	1.689251	-4.56326	-0.43573
H	4.291905	-2.74997	-3.48742
H	3.134339	-4.09006	-3.52919
H	4.44919	-4.09379	-2.33344
H	-6.22249	-1.03044	-3.55843
H	-5.37042	-2.44442	-4.20842
H	-4.73752	-0.81628	-4.51879
H	-5.43591	-3.77776	-1.78493
H	-6.26548	-2.47838	-0.90941
H	-4.79635	-3.22194	-0.2295
H	-1.91014	-1.85091	-3.14633
H	-2.76537	-3.4096	-3.14527
H	-2.04239	-2.79059	-1.64718
H	-4.81806	-1.16296	4.619395
H	-4.11937	-2.79413	4.501302
H	-5.34275	-2.26834	3.326755
H	-3.32859	-3.21415	1.105763
H	-2.25779	-3.65292	2.445925
H	-1.64438	-2.62048	1.147113
H	-1.23923	-1.76306	4.399985
H	-1.72615	-0.06148	4.545898
H	-0.71557	-0.58883	3.174795
H	-1.93685	3.463056	3.278323
H	-1.60457	2.877798	1.63615

H	-1.15466	1.884107	3.034978
H	-4.56234	3.375298	0.846596
H	-4.82589	3.951768	2.500727
H	-5.87592	2.687973	1.834421
H	-4.28898	2.597872	4.859162
H	-3.64845	0.951938	5.027543
H	-5.30231	1.209635	4.417389
H	5.637754	-3.11515	2.848767
H	5.246493	-1.39171	2.687895
H	6.367154	-2.1646	1.54231
H	1.641833	-3.56286	1.696891
H	2.364134	-2.484	2.910126
H	2.874308	-4.18162	2.81806
H	3.903084	-5.07842	-0.27101
H	4.945282	-5.15277	1.163858
H	5.530307	-4.35727	-0.3102
H	3.565713	3.489476	2.259082
H	4.025869	4.59158	0.938653
H	5.210666	4.150294	2.183268
H	5.328389	0.976446	2.764362
H	6.761424	1.901145	2.281448
H	6.419919	0.34636	1.504036
H	7.09192	3.541035	0.066199
H	6.115578	3.424849	-1.41449
H	7.067263	2.029173	-0.85723
H	3.702338	0.315432	-2.39632
H	5.274133	0.960363	-1.96016
H	5.509999	-0.71342	-0.15961
H	5.230906	-1.49994	-1.70326
H	-5.34559	1.46491	-0.29
H	-6.16665	0.007579	-0.81245
H	-5.26238	-1.27895	1.030802
H	-5.89593	0.209562	1.703751

Supplementary References:

1. Sheldrick, G. M. *SHELXT*—Integrated space-group and crystal-structure determination. *Acta Crystallogr.* **A71**, 3–8 (2015).
2. Sheldrick, G. M. A short history of *SHELX*. *Acta Crystallogr*. **A64**, 112–122 (2008).
